# Tau368 improves p-tau diagnostic accuracy for FTLD-tau from FTLD-TDP

**DOI:** 10.1007/s00401-026-03042-1

**Published:** 2026-06-24

**Authors:** Przemysław R. Kac, Katheryn A. Q. Cousins, Alicja Szadziewska, Leslie M. Shaw, Michael Turton, Vivianna M. Van Deerlin, Peter Harrison, Henrik Zetterberg, David A. Wolk, Corey T. McMillan, Douglas Galasko, Jörg Hanrieder, Edward B. Lee, Hlin Kvartsberg, David J. Irwin, Kaj Blennow

**Affiliations:** 1 Department of Psychiatry and Neurochemistry, Institute of Neuroscience and Physiology, The Sahlgrenska Academy at the University of Gothenburg, 431 80 Mölndal, Sweden; 2 Department of Pathology and Laboratory Medicine, School of Medicine and Public Health, University of Wisconsin-Madison, Madison, WI 53726 USA; 3 Department of Neurology, Perelman School of Medicine, University of Pennsylvania, Philadelphia, PA 19104 USA; 4 Department of Pathology and Laboratory Medicine, Perelman School of Medicine, University of Pennsylvania, Philadelphia, PA 19104 USA; 5 Bioventix Plc, Farnham, GU9 7SX UK; 6 Department of Pathology and Laboratory Medicine, Center for Neurodegenerative Disease Research, Institute on Aging, Perelman School of Medicine, University of Pennsylvania, Philadelphia, PA 19104 USA; 7 Clinical Neurochemistry Laboratory, Sahlgrenska University Hospital, 431 80 Mölndal, Sweden; 8 Department of Neurodegenerative Disease, Dementia Research Centre, Institute of Neurology, University College London, Queen Square, London, WC1E 6BT UK; 9 UK Dementia Research Institute, University College London, London, WC1E 6BT UK; 10 Centre for Brain Research, Indian Institute of Science, Bangalore, India; 11 Department of Neurosciences, University of California, San Diego, CA 92161 USA; 12 Eli Lilly and Company, Solna, Sweden

**Keywords:** Frontotemporal lobar degeneration, Alzheimer’s disease, Tau368, p-Tau, Biomarkers, Cerebrospinal fluid

## Abstract

Across tauopathies—Alzheimer’s disease (AD), frontotemporal lobar degeneration due to tau (FTLD-tau)—we compared cerebrospinal fluid (CSF) biomarkers of phosphorylated-tau (p-tau) p-tau181, p-tau212, tau368, total tau (t-tau), and the tau368/t-tau ratio, and tested differentiation from non-tau (FTLD-TDP, neuronal α-synuclein disease (αSyn), and controls). In AD, CSF p-tau181 and p-tau212 were significantly higher than in all other groups, including FTLD-tau, while tau368/t-tau was significantly lower. With the aim to combine tau phosphorylation biomarkers (p-tau181 and p-tau212) with biomarkers for severity of tau pathology (tau368), we made ratios between these biomarkers and examined their diagnostic accuracy in FTLD after excluding high/intermediate AD neuropathologic change (ADNC). CSF p-tau181 and p-tau212, as well as p-tau181/tau368 and p-tau212/tau368, were higher in FTLD-tau compared with non-tau groups, and the diagnostic accuracy to discriminate FTLD-tau from FTLD-TDP improved. Levels of the ratios were increased in behavioral and primary progressive aphasia variants of tauopathies when compared to FTLD-TDP. Furthermore, the biomarkers showed significant correlation with both FTLD-tau and AD tau burden at autopsy across brain regions. These results suggest unique patterns of increased relative levels of p-tau epitopes in CSF among ADNC and FTLD-tau could improve the diagnosis of tauopathies and inform inclusion criteria in clinical trial design.

**Supplementary Information:**

The online version contains supplementary material available at 10.1007/s00401-026-03042-1.

## Main

Tauopathies are an umbrella term for neurodegenerative diseases characterized by abnormalities in the tau protein [73]. Alternative splicing of the *MAPT* gene leads to the translation of multiple tau isoforms, of which six are majorly represented in the brain [27]. Those isoforms could contain inserts of exons 2, 3, and 10. The absence or presence of the latter results in 3 times repeated domain (3R) or 4 times repeated domain (4R) in the microtubule-binding region (MTBR) of tau protein. The most common tauopathy is Alzheimer’s disease (AD) neuropathologic change (ADNC) [19]—a leading cause of dementia, characterized by secondary (downstream) tau pathology composed of both 3R and 4R paired-helical filaments (PHFs) of tau in addition to extracellular amyloid-beta (Aβ) plaque pathology [73]. Several primary tauopathies fall under the heterogeneous umbrella term of frontotemporal lobar degeneration due to tau (FTLD-tau). Those include both sporadic subtypes or those caused by *MAPT* mutation [37, 107]. In contrast to AD [121], there are no robust and reliable fluid biomarkers specific to FTLD-tau [21], which is an obstacle for clinical trials. The great exigency of fluid biomarkers comes from exceptional heterogeneity of FTLD, which can be manifested both clinically—including behavioral frontotemporal dementia (bvFTD) or language variants (primary progressive aphasia; PPA)—and biologically, where aside from tau, TAR-DNA binding protein 43 (TDP-43; FTLD-TDP) inclusions are observed in roughly 60% of patients [37, 39, 54, 63, 76]. Moreover, each proteinopathy group can be further subdivided: [63, 81] including sporadic 4R tauopathies—corticobasal degeneration (CBD), progressive supranuclear palsy (PSP), globular glial tauopathy (GGT), and argyrophilic grain disease (AGD) [8, 71, 106]; sporadic 3R tauopathy of Pick’s disease (PiD); or FTLD-TDP subtypes A–E [76, 82]. Some clinical FTD syndromes are statistically enriched with a greater frequency of specific forms of FTLD, including the semantic variant of PPA (svPPA), which is associated with FTLD-TDP type C in about 80% of cases [38, 93] and also recently found to be an annexin A-11 co-proteinopathy [3, 105]. SvPPA can be less commonly caused by PiD [38] or GGT [36]. Patients who meet formal clinical criteria for PSP [46] are highly likely to have 4R tauopathy, but patients with corticobasal syndrome (CBS) have heterogeneous etiologies [19, 32, 75, 89]. Indeed, CBS and other FTD syndromes with motor features can also be clinically difficult to differentiate from neuronal α-synuclein (αSyn) diseases such as Lewy body dementia [18], where α-synuclein aggregations are observed [115]. Moreover, there is also considerable clinical and pathological overlap with AD, as ADNC can present with FTD-like syndromes [101] and conversely FTLD neuropathology can present with amnestic symptoms [103]. Finally, clinical FTD features often overlap throughout the course of the disease [98]. Thus, clinical diagnosis alone is insufficient for predicting FTLD pathology, and biological markers that reflect underlying FTLD pathology are urgently needed [55].

The natural candidate biomarker for tauopathies is the tau protein. It is known that tau secreted to CSF and other body fluids is cleaved into fragments [92]. The major cleavage sites occur after amino acids 123 and 224 [15], with the tau224 variant generated by calpain 2 cleavage [16], and other sites generated by calpain 1 or calpain 2 are at amino acids 230–231 and 242–244 [14, 30, 84, 102]. These N-terminal to mid-domain fragments contain the phosphorylation sites used in current AD biomarker assays, such as p-tau181, p-tau212, p-tau217, and p-tau231 [4, 24, 34, 35, 64, 65, 67, 68], while MTBR tau fragments are secreted to a much lower extent [15, 92]. We have multiple CSF and plasma immunoassays to measure specific fragments or posttranslational modifications (phosphorylations) of tau that reach very high accuracy in diagnosing AD [40], and the increase of these soluble N- to mid-terminal tau fragments is associated with Aβ pathology [60, 86, 104]. Thus, the magnitude of fold changes is the highest in AD; to a lesser extent, we observe an increase of soluble tau also in tauopathies such as CBD, PiD, or sometimes lower in PSP [12, 51, 56, 57, 87, 90, 122], but the smaller fold-change has made these tau fragments poor diagnostic biomarkers for FTLD-tau.

Certain tau immunoassays target fibrillar tau pathology, aiming to measure differences in the MTBR, which is also the core of aggregated tau. Methods targeting MTBR-tau variants in CSF were found to be associated with tau pathology [10, 47–50, 58, 59, 113]. Aside from association with tau pathology, another possible function of MTBR-tau fragments in CSF was revealed. Levels of MTBR-tau biomarkers, such as the tryptic peptides MTBR-tau275 and MTBR-tau282, and endogenously truncated tau species measured by tau368 targeting immunoassay, were found to remain stable across different neurodegenerative diseases and the AD continuum [48, 59, 113, 114]. When applying these MTBR-tau biomarkers in ratios with N-terminal to mid-domain “total” tau (t-tau) tau biomarkers, performance was high due to the counterbalance between aggregation and total tau secretion status, and likely also for normalizing for inter-individual variability and minimizing preanalytical effects [10, 48, 78, 79]. Considering this unique dual function of tau368 fragment, and having in mind that the amount of aggregated tau protein in brain tissue is much higher [74], and tau protein change in CSF is more prominent [57, 87] in AD than in FTLD-tau we hypothesized that “controlling” for the balance between phosphorylation and MTBR fragments as well as intra-individual variation, may significantly improve the diagnostic performance of the biomarker for FTLD-tau. Since we lack biomarkers to detect FTLD-tau, and ADNC co-pathology influences biomarker values in a minority of those cases [56, 57], examination in rare autopsy cohorts is critically important.

Here, we examined tau368 immunoreactivity in postmortem hippocampus and frontal cortex sections from individuals spanning different stages of AD pathology, as well as from selected individuals with non-AD neurodegenerative diseases, including neuronal α-synuclein disease and FTLD-TDP/hippocampal sclerosis. We used tau368 immunofluorescence together with luminescent conjugated oligothiophenes (LCOs) to compare baseline neuronal tau368 staining, AD-associated aggregate pathology, and tau368 signal in non-AD disease tissue. Next, we evaluated the utility of CSF p-tau181, p-tau212, tau368, t-tau, and their ratios (p-tau181/tau368, p-tau212/tau368, tau368/t-tau) to discriminate postmortem confirmed ADNC from other neurodegenerative diseases in a rare autopsy cohort and healthy participants. We tested CSF biomarker correlation with brain Aβ and tau burden across brain regions. Further to test tau biomarker associations with FTLD-type tau, we continued our analyses in an ADNC-negative subcohort (i.e., FTLD, αSyn, and cognitively normal participants who had not/low ADNC or were CSF Aβ42/Aβ40 negative). We tested the diagnostic accuracy of the p-tau181, p-tau212, tau368, and their ratios in discriminating between FTLD types, and compared levels of the biomarkers between ADNC-negative participants who belonged to the FTLD-tau and FTLD-TDP groups.

## Methods

### UCSD immunohistochemistry brain samples

To characterize the deposition of tau368 in neurofibrillary tangles and assess the conformational states of tau aggregates in Alzheimer’s disease (AD), we performed dual immunofluorescence staining on postmortem human brain sections. We used the tau368 antibody in combination with luminescent conjugated oligothiophenes (LCOs), conformation-sensitive dyes that bind β-sheet-rich structures in both plaques and neurofibrillary tangles. The cohort used for immunohistochemistry consisted of medial frontal cortex (*n* = 15) and hippocampus (*n* = 14) brain samples from (*n* = 16) research participants enrolled in the University of California, San Diego (UCSD) Shiley-Marcos Alzheimer’s Disease Research Center (ADRC). This included one additional normal-control frontal cortex case used as a baseline reference for tau368/LCO staining. Two participants contributed only frontal cortex sections, and one participant contributed only a hippocampus section. Tissue sections from the hippocampus and medial frontal cortex from individuals at varying stages of AD pathology, as well as from individuals with non-tauopathies including αSyn and FTLD-TDP, were analyzed (*n* = 14) (Supplementary Table 1). One slide per region per case was analyzed for immunostaining. Postmortem interval (PMI) data were not available for these cases. RNA integrity number (RIN) was not assessed as this study did not involve RNA-based analyses.

Standardized protocols were used to perform an autopsy [120]. Postmortem neuropathological examination was performed to determine the presence and extent of amyloid and tau pathologies consistent with Alzheimer’s disease, as well as with other neurodegenerative diseases. AD pathology was determined using the CERAD [95], Thal [117], Braak [13], and NIA-Reagan criteria [53] to determine Alzheimer’s disease neuropathologic changes (ADNC).

### Immunohistochemistry

Formalin-fixed paraffin-embedded sections (5 μm thick) were subjected to sequential washes of xylene (2 × 3 min), xylene with 95% ethanol 1:1 (3 min), 95% ethanol (3 min), 70% ethanol (3 min), 50% ethanol (3 min), and Milli-Q water (5 min). Sections underwent heat-induced antigen retrieval in 10 mM sodium citrate buffer (pH 6.0) using a microwave at 100 °C for 20 min. Samples were then washed twice with 1XPBS for 5 min each. To block nonspecific binding, sections were incubated for 90 min with 0.1% normal goat serum diluted in 1XPBS. Primary antibody incubation was performed overnight at 4 °C using an anti-tau368 antibody (mouse monoclonal, in-house, 1:500) diluted in 0.2% PBS-T with 0.01% normal goat serum. Sections were later washed three times in 0.1% PBS-T for 5 min each. Incubation with secondary antibody (Alexa Fluor 647 goat anti-mouse, Invitrogen #A21236) was performed for 60 min at room temperature. Brain sections were treated with autofluorescence quenching agent 1X True Black® for 40 s, followed by 3 washes of 1XPBS, 5 min each. Additionally, sections were co-stained with qFTAA (2.4 μM in 1XPBS) and hFTAA (0.77 μm in 1XPBS) for 25 min at room temperature, followed by a 10-min wash in 1XPBS. Sections were mounted with ProLong™ Gold Antifade Mountant (Invitrogen) and allowed to dry in the dark for 24 h before imaging.

### Microscopy

Multichannel imaging of immuno-stained human brain sections was performed using a high-resolution microscope Olympus VS200 Slide Scanner. Large multi-channel tile scans were captured using FITC and Cy5 filter settings. All the images were captured using a UPLXAPO 20x/0.80 objective lens. All images were captured with the same acquisition parameters. Quantification was performed on raw dual-stained images from the medial frontal cortex and hippocampal CA1. Cases were grouped by Braak stage (I–II, III–IV, and V–VI), and tau368 immunoreactivity was quantified as the tau368-positive area fraction within each region, with individual cases used as the unit of analysis. Positive signal was determined by thresholding in QuPath v0.7.0. The analyzed tissue area per case ranged from 0.54 to 3.21 mm^2^ in medial frontal cortex and from 0.29 to 3.27 mm^2^ in hippocampal CA1. All image analyses were performed blinded to diagnosis and Braak stage.

### Penn biomarker cohort participants

Available CSF samples (*n* = 176) were analyzed from participants from the University of Pennsylvania (Penn) Integrated Neurodegenerative Disease Biobank and Database (INDD) who were followed for observational research through the clinical cores at the Penn ADRC and FTD Center, and Parkinson’s and Movement Disorders Center of Excellence [119]. Lumbar puncture to collect CSF was part of routine research program following standardized collection, processing and storage procedures (− 80 °C) [111]. Inclusion criteria were a neuropathological diagnosis of AD, FTLD-tau, FTLD-TDP, or αSyn and banked CSF; healthy controls were included as a reference group. Written informed consent of participants was obtained and approved by the Penn Institutional Review Board.

Tauopathies were AD and FTLD-tau. AD was determined at autopsy based on established criteria [96] defined as high or intermediate AD neuropathological change (ADNC). FTLD-tau was determined at autopsy by the presence of tau inclusions using neuropathologic criteria [23]; neuropathological diagnoses included Pick’s disease (PiD), progressive supranuclear palsy (PSP), corticobasal disease (CBD), argyrophilic grain disease (AGD), and globular glial tauopathy (GGT). Non-tauopathies were FTLD-TDP, αSyn, and healthy controls. FTLD-TDP included types A–E [82], ALS with untypable TDP-43. αSyn included brainstem, limbic, or diffuse/neocortical Lewy bodies with clinical PD, PDD, or DLB. Healthy controls were without cognitive impairment (Mini Mental State Exam [MMSE] ≥ 28) and were negative for AD by biomarkers (i.e., CSF Aβ42/40 > 0.072[31]). Autopsy data were not available for healthy controls. FTD patients were genotyped for main causative genes for FTLD (i.e., *MAPT, GRN, C9orf72, *etc.) based on risk from structured pedigree analysis, as described. We performed sensitivity analyses of subgroups of FTLD (see below), including focused analysis of FTLD and αSyn with negligible ADNC co-pathology defined by exclusion of FTLD and αSyn patients who met neuropathological criteria for clinically-relevant medium or high-level ADNC [96].

Postmortem brain Aβ and tau pathology burden was scored using immunohistochemistry data that were scored from brain tissue stained on a semiquantitative scale (0 = none, 0.5 = rare, 1 = minimal, 2 = moderate, and 3 = severe) across regions standardly sampled, as previously described [96]. Average brain Aβ and tau was calculated using mean score across sampled regions: amygdala, cingulate, CA1/subiculum, entorhinal cortex, middle frontal gyrus, angular gyrus, superior/middle temporal gyrus, pons, and medulla. PHF-1 antibody was used to quantify the average brain tau metric. Of autopsy cases (*n* = 124), average brain tau data were missing for four AD, one FTLD-tau, and two FTLD-TDP; average brain Aβ data were missing for five AD, zero FTLD-tau, two FTLD-TDP, and zero αSyn.

### Biomarker measurements

P-tau212 and tau368 biomarker assays were performed on the Simoa HD-X platform at the University of Gothenburg as in previously published articles [59, 64]. CSF p-tau212 was measured using published validated in-house immunoassay. Briefly, p-tau212 specific antibody is paired with N-terminal targeting Tau12 antibody (BioLegend). For tau368, we use in-house antibody targeting sequences from both R3/R4 exons in MTBR region of tau as detection, with in-house tau368 specific antibody as capture. In vitro phosphorylated recombinant full-length tau-441 (#269022, Abcam) was used as the assay calibrator for p-tau212 immunoassay, and in-house recombinant Tau1-368 protein was used as the assay calibrator for tau368 immunoassay. An on-plate standard curve was shared for all plates within the study to maintain the accuracy of the measurements. For p-tau212, samples were diluted 10 times prior to measurement, and Tau 2.0 Sample Diluent (#101556, Quanterix, MA, USA) was used as sample diluent. Tau368 samples were run neat. Sample analysis was performed in singlicates with internal quality controls (iQC) CSF samples measured at the start and the end of each technical run. CSF tau368 samples were normalized to a mean of the iQC. The coefficients of variation (CV) of these iQC samples are: 3.9–5% for within-run and 11.3–13.6% for between-run precision for p-tau212 and 6% for both within- and between-runs for tau368. CSF t-tau was analyzed at the Penn Biomarker Research Laboratory using the Luminex xMAP platform, as previously described [110].

For a subset of individuals, CSF Aβ42/Aβ40 (*n* = 132; 34 AD, 20 FTLD-tau, 17 FTLD-TDP, 19 αSyn, 42 Control), and p-tau181 (*n* = 130; 33 AD, 20 FTLD-tau, 17 FTLD-TDP, 18 αSyn, 42 Control) measurements were available, analyzed using the Fujirebio Lumipulse G1200 platform [31] at the Penn Biomarker Research Laboratory.

### Antibody development

Generation of sheep monoclonal antibodies for p-tau212 was undertaken according to the UK Animal Scientific Procedures Act, and the methodology has been described previously [100]. Briefly, custom-designed p-tau peptides phosphorylated at threonine 212 with C-terminal tetanus toxin sequences were synthesized (Severn Biotech, UK). These peptides were used for the immunization of sheep and the monoclonal antibody generation process followed as described [100]. Afterward, candidate hybridomas were selected based on binding to specifically phosphorylated peptides. The p-tau212 demonstrated the highest level of specificity for the p-tau212 epitope. Antibody design, generation and validation were performed at Bioventix Plc (Surrey, United Kingdom).

For tau368 antibody, a new polyclonal antibody specific against tau truncated at aa 368 was generated by immunizing rabbits with 200 µg of a peptide containing the tau360–368 sequence (Caslo ApS, Denmark), in complete Freund’s adjuvant (Sigma). Following one more dose of the immunogen (200 µg/mouse) in Freund’s Incomplete adjuvant (Sigma), and furthermore two more doses of the immunogen (100 µg/mouse) in Freund’s Incomplete adjuvant (Sigma) the rabbits were sacrificed, and standard procedures of antibody generation followed. Antibody validation data by mass spectrometry is shown in the supplementary material in the previously published article [59].

For the tau368 immunoassay detection antibody, the new library of anti-tau mAbs was generated by immunizing 8-week-old Balb/c mice with 100 µg of the recombinant tau241–441 peptide in complete Freund’s adjuvant (Sigma). Following 2–3 further dosages of the immunogen (100 µg/mouse) in Freund’s Incomplete adjuvant (Sigma), the mice were sacrificed, the spleen removed, and B cells fused with the SP2/0 myeloma cell line following standard protocols. Approximately 10 days post-fusion, direct enzyme-linked immunosorbent assay (ELISA) experiments were performed to screen the cell media for antibodies that react with full-length recombinant tau1–441 (2N4R) or tau241–441. Positive clones were further grown, subcloned, and subsequently frozen in liquid nitrogen. Antibody specificity was verified, and the isotype determined using the Pierce Rapid Isotyping Kit-Mouse. Thereafter, the mAbs were purified using a Hitrap protein G column (Cytiva) following the manufacturer’s instructions. Validation of antibodies was presented in previously published articles [58, 59].

### Ethical clearance

Patient participation was performed after informed consent was obtained according to the Declaration of Helsinki and approved by the University of Pennsylvania Institutional Review Board. The UCSD-Neuropathology cohort was reviewed and approved by the human subject review board at UCSD. Informed consent was obtained from all patients or their caregivers consistent with California State law.

### Statistical analyses

Demographic and CSF variables were not normally distributed; Kruskal–Wallis and Mann–Whitney–Wilcoxon tests performed unadjusted group comparisons for continuous variables, Spearman correlations tested two continuous variables, and chi-square tests compared categorical variables. In logistic models, CSF biomarkers were log-transformed and scaled to allow for comparison of β-coefficients and hazard rate (HR). In AD analysis, age at CSF, sex, and APOE ε4 were included as covariates. In FTLD analysis, age at CSF and sex were included as covariates. Missing data were dropped from the models listwise. Median fold change was calculated as the ratio between two groups. Models were two-sided and used a significance threshold of *α* = 0.05, and false detection rate (FDR)-correction accounted for multiple comparisons of seven CSF biomarkers (p-tau181, p-tau212, tau368, t-tau, p-tau181/tau368, p-tau212/tau368, and tau368/t-tau). Receiver operating characteristic (ROC) analysis [118] with bootstrapping (2000 iterations) tested biomarker discrimination of AD from all non-tau, FTLD-tau from all non-tau (excluding AD), and FTLD-tau from FTLD-TDP. Optimal cutpoints were identified at maximum Youden’s, accounting for midpoints. All models were performed in the full group with all participants. To test associations with FTLD-tau, models were repeated after excluding high or intermediate ADNC (ADNC-negative Penn subcohort). Finally, to investigate biomarker associations with FTLD-tau pathology, Pearson’s *r* correlations tested how each biomarker associated with average brain tau burden in the ADNC-negative Penn subcohort and within FTLD-tau. Within FTLD-tau, Hittner’s *Z* compared magnitude of correlations between p-tau isoforms normalized for tau368 (CSF p-tau181/tau368, p-tau212/tau368) vs. t-tau (CSF p-tau181/t-tau, p-tau212/t-tau).

## Results

### Participants

The UCSD cohort used for immunohistochemistry consisted of frontal cortex (*n* = 15) and hippocampus (*n* = 14) brain samples. Tissue sections from the hippocampus and frontal cortex from individuals at varying stages of AD pathology, as well as from individuals with non-tauopathies including αSyn and FTLD-TDP, and one normal-control case, were analyzed (Supplementary Table 1).

The Penn biomarker cohort consisted of individuals with pathological diagnoses (autopsy- or genetically-confirmed) of AD, FTLD-tau, FTLD-TDP, or αSyn and banked CSF (*n* = 176); healthy controls were included as a reference group (Table 1). To test biomarker associations with FTLD-tau pathology, analyses were conducted in the ADNC-negative subcohort (*n* = 106) (Supplementary Table 2).

**Table 1:** Demographic characteristics of the CSF biomarker Penn cohort

	Normal	AD	FTLDtau	FTLDTDP	αSyn	αSyn + AD	*p*	Missing
*n*	41	60	24	30	12	9		
Age at onset (years)	–	67.0 [58.0, 73.2]	65.0 [59.0, 70.0]	60.0 [55.0, 66.0]	61.5 [57.0, 66.0]	65.0 [59.0, 69.0]	0.034	42
Age at CSF (years)	66.0 [61.0, 70.0]	71.0 [62.0, 77.0]	69.5 [62.5, 72.0]	64.0 [58.2, 68.8]	71.0 [67.2, 78.2]	70.0 [63.0, 71.0]	0.008	–
Age at death (years)	76.0 [76.0, 76.0]	78.0 [68.8, 84.2]	72.0 [66.0, 76.5]	67.0 [60.8, 73.2]	78.5 [73.2, 81.0]	75.0 [70.0, 79.0]	0.003	43
CSF to death (years)	6.0 [6.0, 6.0]	6.0 [4.0, 8.2]	4.0 [2.0, 5.5]	4.0 [2.8, 5.0]	4.5 [1.8, 6.2]	5.0 [3.0, 8.0]	0.002	43
Sex = Male (%)	13 (31.7%)	34 (56.7%)	13 (54.2%)	17 (56.7%)	11 (91.7%)	8 (88.9%)	0.001	–
Self-reported Race (%)							0.052	–
Asian	0	0	0	1 (3.3%)	0	0		
Black or African American	9 (22.0%)	3 (5.0%)	0	1 (3.3%)	0	0		
More than one Race	2 (4.9%)	2 (3.3%)	0	1 (3.3%)	0	0		
White	30 (73.2%)	55 (91.7%)	24 (100.0%)	27 (90.0%)	12 (100.0%)	9 (100.0%)		
ClinicalDx (%)							< 0.001	–
Normal	41 (100.0%)	0	0	0	0	0		
MCI	0	10 (16.7%)	0	0	0	0		
Amnestic	0	43 (71.7%)	2 (8.3%)	0	1 (8.3%)	2 (22.2%)		
CBS	0	0	5 (20.8%)	2 (6.7%)	0	1 (11.1%)		
lvPPA	0	2 (3.3%)	2 (8.3%)	0	0	0		
naPPA	0	0	2 (8.3%)	2 (6.7%)	0	0		
svPPA	0	0	0	1 (3.3%)	0	0		
bvFTD	0	3 (5.0%)	10 (41.7%)	16 (53.3%)	0	0		
ALS	0	0	0	5 (16.7%)	0	0		
PSP	0	0	3 (12.5%)	0	0	0		
PD/PDD	0	0	0	0	10 (83.3%)	4 (44.4%)		
DLB	0	0	0	0	1 (8.3%)	2 (22.2%)		
Vascular	0	0	0	1 (3.3%)	0	0		
Dementia NOS	0	2 (3.3%)	0	3 (10.0%)	0	0		
Mutation (%)							< 0.001	–
None	41 (100.0%)	59 (98.3%)	18 (75.0%)	14 (46.7%)	12 (100.0%)	9 (100.0%)		
C9orf72	0	0	0	10 (33.3%)	0	0		
GRN	0	0	0	5 (16.7%)	0	0		
MAPT	0	0	6 (25.0%)	0	0	0		
TARDBP	0	0	0	1 (3.3%)	0	0		
PSEN1	0	1 (1.7%)	0	0	0	0		
ADNC (%)							–	53
Not	–	0	6 (28.6%)	10 (47.6%)	3 (25.0%)	0		
Low	–	0	15 (71.4%)	10 (47.6%)	9 (75.0%)	0		
Intermediate	–	3 (5.0%)	0	1 (4.8%)	0	8 (88.9%)		
High	–	57 (95.0%)	0	0	0	1 (11.1%)		
Brain Aβ burden	–	2.3 [2.2, 2.4]	0.5 [0.1, 1.0]	0.1 [0.0, 1.2]	0.4 [0.1, 1.2]	1.9 [1.7, 2.3]	< 0.001	59
Brain tau burden	–	2.5 [2.2, 2.6]	2.0 [1.8, 2.4]	0.6 [0.2, 1.0]	0.7 [0.6, 1.1]	1.4 [1.0, 1.9]	< 0.001	59
CSF p-tau181	28.2 [23.1, 32.8]	95.8 [68.8, 131.3]	40.5 [30.8, 47.1]	28.9 [23.1, 32.8]	25.8 [21.9, 31.6]	45.8 [30.7, 54.7]	< 0.001	38
CSF t-tau	44.0 [37.0, 57.7]	91.1 [68.2, 150.2]	56.0 [45.8, 79.2]	60.0 [45.2, 85.2]	36.0 [26.5, 49.0]	53.0 [47.3, 79.0]	< 0.001	–
CSF p-tau212	4.4 [3.1, 7.0]	37.5 [26.1, 50.3]	7.6 [5.1, 12.9]	3.7 [2.2, 4.9]	3.8 [3.0, 4.6]	10.0 [6.7, 23.4]	< 0.001	3
CSF tau368	17.6 [14.8, 19.8]	17.2 [13.2, 24.2]	15.7 [13.4, 20.3]	16.4 [13.8, 21.7]	13.8 [10.3, 17.7]	16.8 [15.6, 22.0]	0.527	6
CSF p-tau212/tau368	0.3 [0.2, 0.4]	2.0 [1.3, 2.9]	0.6 [0.3, 0.8]	0.2 [0.2, 0.3]	0.3 [0.2, 0.4]	0.7 [0.4, 1.6]	< 0.001	9
CSF p-tau181/tau368	1.5 [1.3, 2.0]	5.8 [4.1, 6.8]	2.5 [2.1, 2.8]	1.8 [1.6, 2.0]	2.1 [1.3, 2.5]	2.9 [1.9, 3.3]	< 0.001	44
CSF Aβ42/Aβ40	0.098 [0.094, 0.103]	0.043 [0.037, 0.051]	0.091 [0.081, 0.095]	0.090 [0.078, 0.096]	0.094 [0.088, 0.100]	0.062 [0.055, 0.066]	< 0.001	38
CSF tau368/t-tau	0.378 [0.327, 0.466]	0.179 [0.149, 0.235]	0.287 [0.216, 0.366]	0.284 [0.238, 0.330]	0.381 [0.314, 0.581]	0.286 [0.250, 0.360]	< 0.001	6
CSF p-tau212/t-tau	0.104 [0.084, 0.135]	0.362 [0.298, 0.453]	0.160 [0.097, 0.218]	0.064 [0.044, 0.078]	0.117 [0.089, 0.159]	0.255 [0.142, 0.425]	< 0.001	3
CSF p-tau181/t-tau	0.614 [0.532, 0.680]	1.031 [0.882, 1.183]	0.605 [0.568, 0.810]	0.496 [0.387, 0.640]	0.708 [0.592, 0.971]	0.791 [0.633, 0.997]	< 0.001	38

*Aβ* amyloid-β, *ALS* amyotrophic lateral sclerosis, *AD* Alzheimer’s disease, *ADNC* Alzheimer’s disease neuropathologic change, *bvFTD* behavioral variant Frontotemporal Dementia, *CSF* cerebrospinal fluid, *C9orf72* chromosome 9 open reading frame 72, *normal* clinically healthy individuals without cognitive impairment, *CBS* corticobasal syndrome, *DLB* dementia with Lewy bodies, *FTLD-tau* frontotemporal lobar degeneration type tau, *FTLD-TDP* frontotemporal lobar degeneration type TDP, *GRN* granulin, *αSyn* Lewy body disease with alpha-synuclein/Parkinson’s Disease, *αSyn + AD* Lewy body disease with alpha-synuclein/Parkinson’s Disease with concominant AD co-pathology, *lvPPA* logopenic variant primary progressive aphasia, *MAPT* microtubule-associated protein Tau, *PD/PDD* Parkinson’s disease/Parkinson’s disease dementia, *p-tauX* phosphorylated-tauX, *PSEN* Presenilin1, *PSP* progressive supranuclear palsy, *svPPA* semantic variant primary progressive aphasia, *t-tau* total tau

### Immunofluorescence staining of tau368 and luminescent conjugated oligothiophenes (LCOs) in human post-mortem brain tissue

Previous work demonstrated tau368 immunoreactivity in normal-control brain, where staining was observed primarily as neuronal nuclear and cytoplasmic signal, as well as stronger neuronal and filamentous staining in AD brain [10]. To define the neuropathological pattern of tau368 immunoreactivity, we performed tau368/LCO double staining in postmortem brain tissue from AD cases at different Braak stages, selected non-AD neurodegenerative disease cases, and a normal-control frontal cortex sample.

**Fig. 1:**
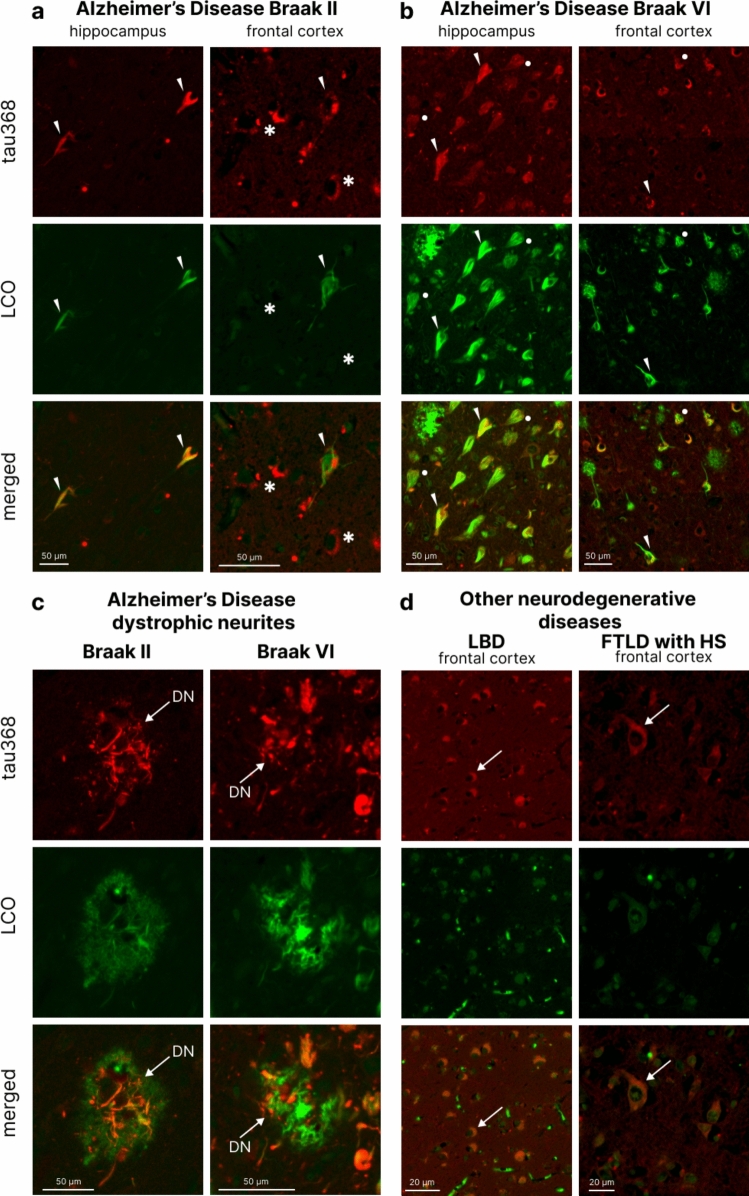
Immunofluorescence staining of tau368 and luminescent conjugated oligothiophenes (LCOs) in human postmortem brain tissue from UCSD cohort. **a** Asterisks indicate pretangles; white arrowheads indicate mature neurofibrillary tangles; circles indicate extracellular ghost tangles; and yellow dashed rectangles indicate representative areas of weak tau368 immunoreactivity in non-AD disease cases. Hippocampal and frontal cortex sections from individuals with Alzheimer’s disease at Braak stage II exhibit a low burden of tau pathology. tau368 staining reveals a granular staining pattern consistent with early tau aggregation, including both pretangles (examples marked with asterisks) and occasional mature neurofibrillary tangles (examples marked with arrowhead). In the frontal cortex, staining primarily reflects pretangles. LCO staining, which binds to β-sheet-rich protein aggregates, highlights these tau inclusions, indicating conformationally altered tau structures. **b** Alzheimer’s disease Braak stage VI hippocampal and frontal cortex sections exhibit a high density of tau pathology. tau368 shows intense accumulation within neuronal somata. LCO staining reveals fibrillar aggregates, with strong co-localization with tau368, particularly in mature (examples marked with arrowhead) and extracellular ghost tangles (examples marked with a circle). **c** Neuritic plaques are observed in Alzheimer’s disease cases at Braak stages II and VI. They exhibit LCO staining indicative of β-sheet-rich amyloid structures, a subset of these plaques contains dystrophic neurites (marked with arrow) that are immunoreactive for tau368, indicating tau accumulation within neuritic processes. **d** Frontal cortex tissue from other neurodegenerative diseases, including neuronal α-synuclein disease (Lewy body dementia limbic (transitional) type—LBD) and frontotemporal dementia (FTD) with hippocampal sclerosis, displays sparse moderate tau368 immunoreactivity (marked with yellow rectangles). LCO staining is minimal, indicating the absence of mature tau fibrils in these samples. Immunofluorescence was performed on hippocampus (*n* = 14) and frontal cortex (*n* = 14) tissue sections. Images shown are representative of pathology patterns observed across individuals. Scale bars: 50 µm (**a**–**c**), 20 µm (**d**)

In early-stage AD cases (Braak stage II, Fig. 1a), both hippocampal and frontal cortex sections displayed low burden of tau pathology. The tau368 immunoreactivity was observed in individual neurons, presenting as small, granular deposits within the cytoplasm. Staining revealed mostly pretangles and occasional mature neurofibrillary tangles, reflecting an early stage of tau aggregation with limited progression to more advanced fibrillar inclusions. LCO labeling in these sections highlighted a subset of early tau aggregates with conformationally altered tau. In contrast, brain tissue from individuals at advanced disease stages (Braak stage VI, Fig. 1b) demonstrated an increase in tau pathology, with widespread and prominent tau368 staining observed both in the hippocampus and frontal cortex. At this advanced stage, dense intracellular labeling of tau368 was observed, with tau aggregates forming compact and well-defined neurofibrillary tangles. In addition, extracellular ghost tangles, consisting of tau-rich remnants of degenerating neurons, were frequently observed. In the frontal cortex, pretangles were commonly observed as round cells with tau368 deposition. LCO staining was robust and extensively colocalized with tau368, indicating a high burden of conformationally altered, fibrillar tau structures associated with advanced neurodegenerative changes. Quantification of tau368-positive area fraction supported these qualitative observations (Supplementary Fig. 1). In the frontal cortex, tau368-positive area fraction was significantly higher in Braak V–VI than in Braak I–II. In hippocampal CA1, tau368-positive area fraction was significantly higher in Braak V–VI than in both Braak I–II and Braak III–IV, consistent with progressive accumulation of tau368 with advancing neurofibrillary pathology. Neuropil threads, which are tau-positive structures found in the axonal and dendritic processes, did not exhibit detectable tau368 immunoreactivity throughout the sample in the examined cases, suggesting that tau aggregation in these cases was predominantly confined to the cell bodies, rather than the finer axonal structures.

Additionally, in AD cases, we observed plaques with dystrophic neurites positive for tau368 (Fig. 1c). Neuritic plaques were observed at both Braak stage II and VI with varied frequency that reflects the pathology progression. These plaques with high β-amyloid content are recognized by LCOs staining. Within a subset of plaques, tau368 immunoreactivity was observed in dystrophic neurites, indicating that tau aggregation extends into neuritic processes.

Finally, we assessed tau368 immunoreactivity in selected non-AD neurodegenerative disease cases, including αSyn and FTLD with hippocampal sclerosis (Fig. 1d). In these sections, tau368 labeling was sparse and the observed signal was moderately stronger than the baseline neuronal tau368 immunoreactivity observed in normal-control tissue (Supplementary Fig. 2), with absent LCO colocalization. In these samples, we also observed occasional LCO-positive, tau368-negative aggregates, as LCO labels β-sheet-rich aggregates without defining protein identity, these structures were not assigned to a specific molecular pathology in this experiment.

### Diagnostic performance of biomarkers to distinguish autopsy-verified AD from other neurodegenerative diseases

Logistic regression tested how biomarkers (log-transformed and scaled) discriminated AD from all other participants, covarying for age, sex, and APOE ε4. In order of absolute largest HR to smallest, CSF p-tau181/tau368 (HR 8223.7, *β* = 9, 95% CI 4.9–17, *p* = 0.0021, FDR-*p* = 0.014), p-tau212/tau368 (HR 36.6, *β* = 3.6, 95% CI 2.5–5.1, *p* = 3.2e−08, FDR-*p* = 2.2e−07), p-tau181 (HR 30.0, *β* = 3.4, 95% CI 2.3–4.9, *p* = 2.5e−07, FDR-*p* = 1.7e−06), and p-tau212 (HR 16.4, β = 2.8, 95% CI 2–3.8, *p* = 5.7e−10, FDR-*p* = 4e−09) predicted AD vs all other participants, and outperformed CSF tau368/t-tau (HR 0.10, *β* = − 2.4869, 95% CI − 3.4 to − 1.7, *p* = 6.4e−09, FDR-*p* = 4.5e−08) and t-tau (HR 1.00, *β* = 0.0069, 95% CI 0.0043–0.01, *p* = 3.2e−06, FDR-*p* = 0.000022). CSF tau368 was not significant (*p* = 1). On the contrary to the p-tau/tau368 ratio, p-tau/t-tau ratios decreased the diagnostic performance of p-tau biomarkers (Supplementary Fig. 3A).

**Fig. 2:**
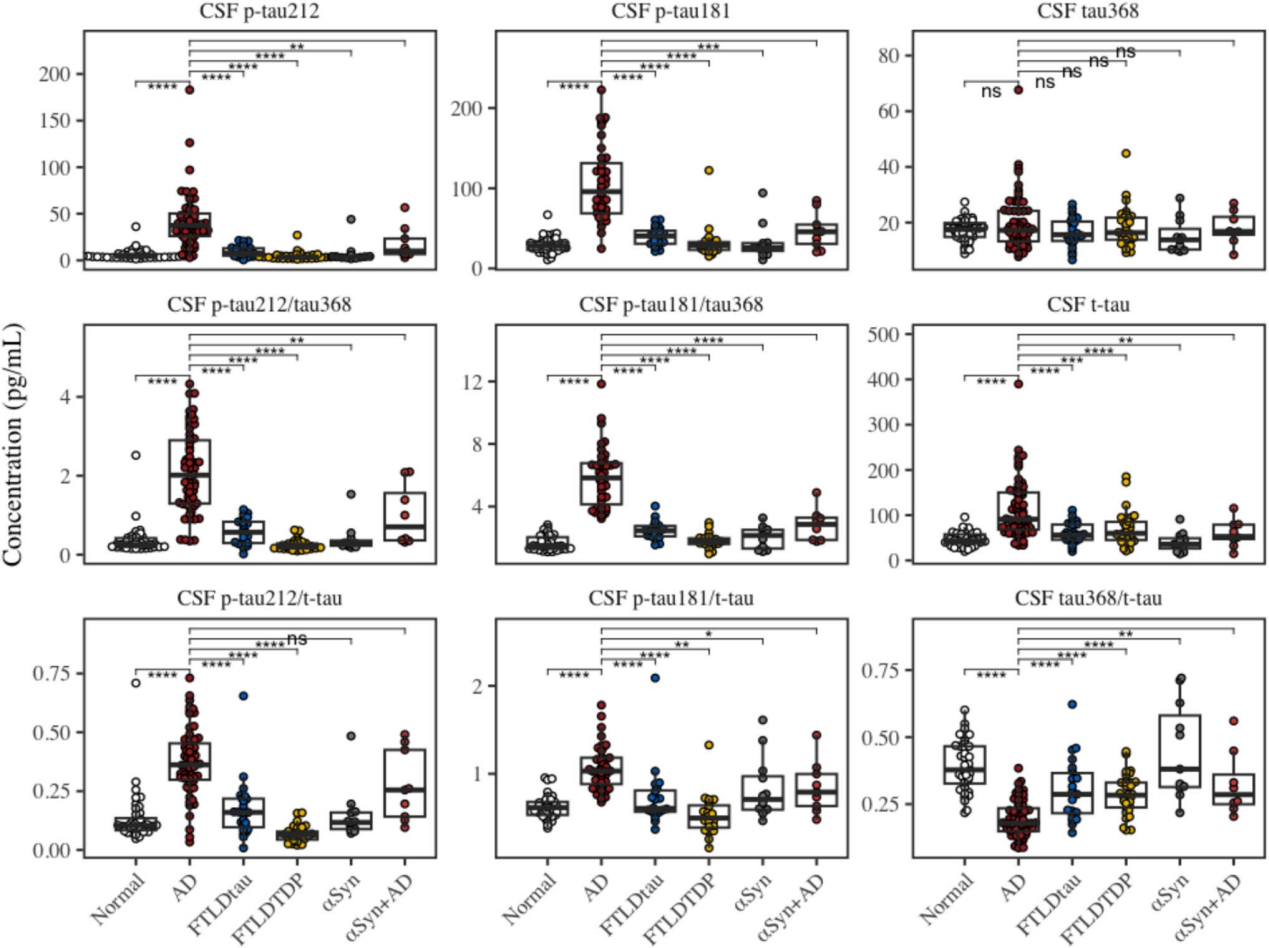
Levels of tau biomarkers across the Penn cohort. The figure shows levels of tau biomarkers in clinically healthy individuals without cognitive impairment (normal; *n* = 41), Alzheimer’s disease (AD; *n* = 60), frontotemporal lobar degeneration type tau (FTLD-tau; *n* = 24), frontotemporal lobar degeneration type TDP (FTLD-TDP; *n* = 30), Lewy body disease with alpha-synuclein (αSyn; *n* = 12), and Lewy body disease with alpha-synuclein with concomitant AD co-pathology (αSyn + AD; *n* = 9). Box plots represent the median and interquartile range (IQR), and the boundaries of the whiskers are the minimum to maximum values. The Wilcoxon test is used to calculate differences across groups. *; **; ***; **** represent *p* < 0.05; *p* < 0.01; *p* < 0.001; *p* < 0.0001 respectively

Pairwise comparisons between AD and the other group for all CSF biomarkers and their respective ratios are in Fig. 2. Focusing on p-tau212/tau368 and p-tau181/tau368, which had the highest HRs, we computed pairwise median fold change. In reference to healthy controls, fold change in AD was 7.83× for CSF p-tau212/tau368 and 3.90× for p-tau181/tau368. In AD vs. FTLD-tau, fold change was 3.55× for CSF p-tau212/tau368 and 2.38× for p-tau181/tau368. In AD vs. FTLD-TDP, fold change was 9.84×  for CSF p-tau212/tau368 and 3.32× for p-tau181/tau368. In AD vs. αSyn, fold change was 7.43× for CSF p-tau212/tau368 and 2.72× for p-tau181/tau368. In AD vs. αSyn + AD fold change was 2.86× for CSF p-tau212/tau368 and 2.04× for p-tau181/tau368.

### Correlation of biomarkers in the whole Penn cohort with core CSF AD biomarkers and ADNC in the brain

For all tau biomarkers, except tau368 alone, we observed moderate correlations with CSF Aβ42/Aβ40 ratio (absolute rho = 0.51–0.71) (Supplementary Fig. 4). Tau368, similar to previous publications, correlated with CSF t-tau, p-tau181, and p-tau212, confirming the association with soluble tau (Supplementary Figs. 4, 5, 6). Significant correlations for p-tau and t-tau biomarkers with each other were observed (rho = 0.72–0.92) (Supplementary Fig. 4). Regarding ADNC, moderate to strong correlations were also observed for Thal phase, Braak Staging for all the biomarkers but tau368; for the latter biomarker, only weak correlations were observed (Supplementary Fig. 4). To further evaluate if tau biomarkers were associated with postmortem tau pathology in the brain, we performed regional pair-wise correlations with postmortem pathology. A semi-quantitative 5-point scale (0 = none to 3 = severe) was used to assess brain tau burden and Aβ; a composite metric averaged postmortem tau burden and Aβ burden over sampled regions. CSF biomarkers had a wide range of correlations with tau burden across different brain regions. However, tau368 was only correlated with tau burden in the motor cortex and spinal cord. p-tau181 and p-tau212 correlations with average brain tau burden increased when we used the ratio with tau368 (Supplementary Figs. 5, 6). Correlation with CSF Aβ42/Aβ40 ratio or postmortem pathology was relatively weaker for p-tau/t-tau ratio when compared to biomarkers alone, or p-tau/tau368 ratio (Supplementary Figs. 4, 5). Together, those results verify associations of CSF antemortem tau biomarkers with tau pathology in the brain, and confirm that CSF Aβ42/Aβ40 ratio reflects amyloid burden in the brain, aligning with the results from previously published studies [40, 42, 64].

### CSF p-tau/tau368 ratios improve the diagnostic accuracy of p-tau biomarkers in discriminating FTLD-tau from FTLD-TDP

Having confirmed associations of CSF biomarkers with ADNC, we tested associations with FTLD-tau. FTLD-TDP, αSyn, and healthy controls were included with no/low ADNC or CSF Aβ42/Aβ40 > 0.072 (ADNC-negative subcohort; *n* = 106). Analyses revealed that FTLD-tau PSP cases were outliers for tau368 ratio levels (Supplementary Fig. 10), and that PSP had low brain tau burden (median = 1.47) compared to other FTLD-tau subtypes (CBD = 2.44; Tau untypable = 2.22; PiD = 1.94; GGT = 1.89; AGD = 1.5). Thus, analyses in ADNC-negative subcohort excluded four PSP cases (*n* = 102). Analyses including PSP are reported in supplementary material (Supplementary Fig. 10; Supplementary Tables 4–5).

Logistic regression tested how biomarkers (log-transformed and scaled) discriminated FTLD-tau from non-tau (FTLD-TDP, αSyn, Controls), covarying for age and sex. In order of largest to smallest HR, CSF p-tau181/tau368 (HR 8.9, *β* = 2.2, 95% CI 1.2–3.7, *p* = 0.00051, FDR-*p* = 0.0036), p-tau212/tau368 (HR 4.0, *β* = 1.4, 95% CI 0.78–2.1, *p* = 0.000048, FDR-*p* = 0.00034), p-tau181 (HR 3.8, *β* = 1.3, 95% CI 0.61–2.2, *p* = 0.001, FDR-*p* = 0.007), and p-tau212 (HR 3.5, *β* = 1.3, 95% CI 0.68–2, *p* = 0.0001, FDR-*p* = 0.0007) predicted FTLD-tau vs non-tau, and outperformed t-tau (HR 1.00, *β* = 0.0062, 95% CI 0.0022–0.011, *p* = 0.0034, FDR-*p* = 0.024). CSF tau368 was not significant (*p* = 0.36) and CSF tau368/t-tau did not survive FDR-correction (HR 0.50, *β* = − 0.7809, 95% CI − 1.4 to − 0.24, *p* = 0.0075, FDR-*p* = 0.052). In addition, we tested p-tau/t-tau ratios, since p-tau181/t-tau had previously been proposed as a biomarker of FTLD-tau. CSF p-tau181/t-tau was not significant (HR 3.3, *β* = 1.2, 95% CI − 0.7–3, *p* = 0.2, FDR-*p* = 1) and p-tau212/t-tau did not survive FDR-correction (HR 504.3, *β* = 6.2, 95% CI 1.8–12, *p* = 0.013, FDR-*p* = 0.089) (Supplementary Fig. 9).

**Fig. 3:**
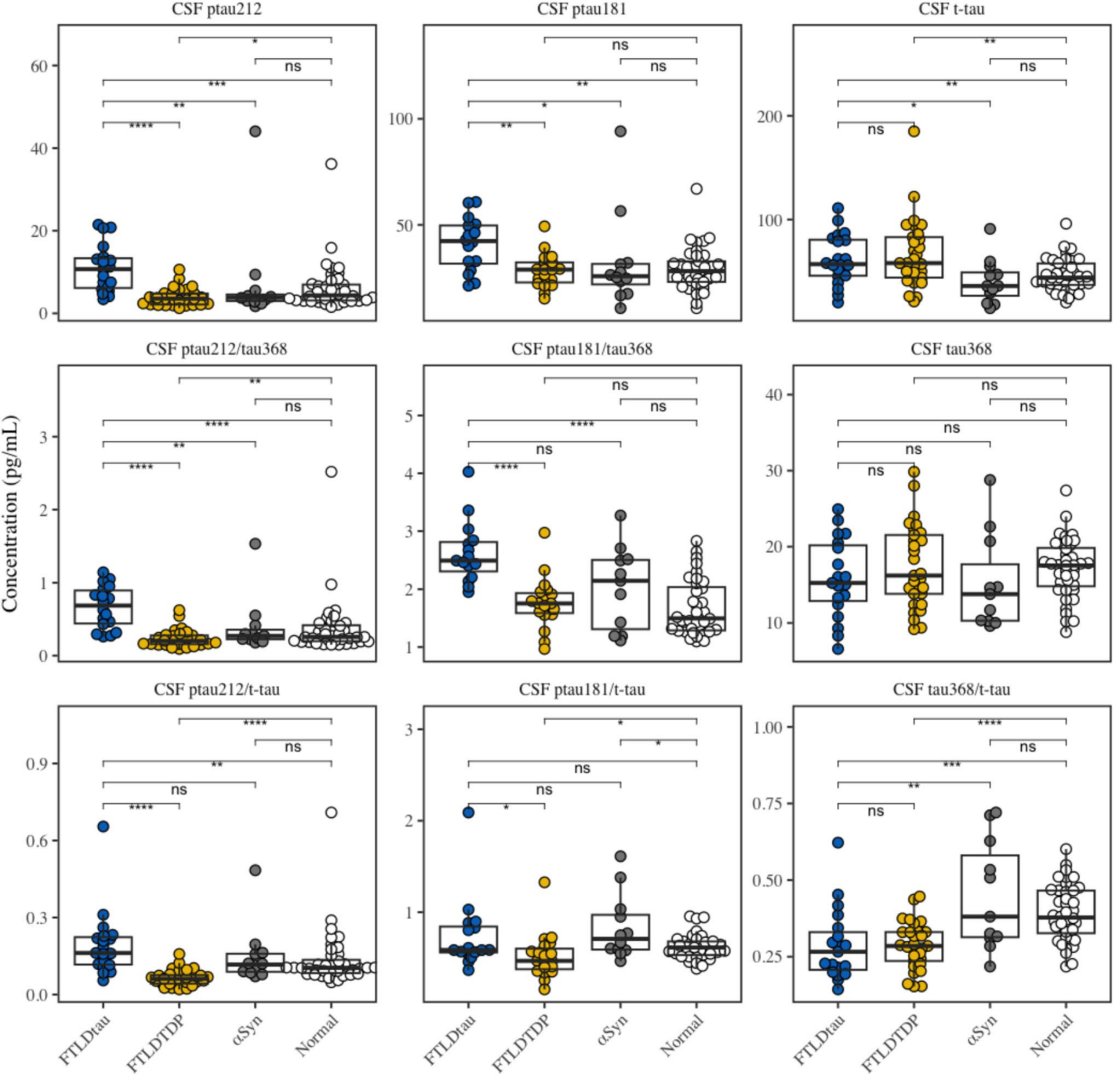
Levels of biomarkers in ADNC-negative Penn subcohort. The figure shows levels of tau biomarkers in clinically healthy individuals without cognitive impairment (normal; *n* = 41), frontotemporal lobar degeneration type tau (FTLD-Tau; *n* = 21), frontotemporal lobar degeneration type TDP (FTLD-TDP; *n* = 21), and neuronal α-synuclein diseases (αSyn; *n* = 12). Box plots represent median and interquartile range (IQR), and boundaries of the whiskers are minimum to maximum values. Wilcoxon test is used to calculate differences across groups. *; **; ***; **** represent *p* < 0.05; *p* < 0.01; *p* < 0.001; *p* < 0.0001 respectively

Pairwise comparisons between FTLD-tau and non-tau for all CSF biomarkers and their respective ratios are in Fig. 3. The highest median fold change was observed for p-tau212/tau368 ratio when FTLD-tau was compared to FTLD-TDP (3.38×), followed by p-tau212 alone (3.01× median fold change); by comparison, median fold changes were smaller for p-tau181/tau368 (1.42×) and p-tau181 (1.47×). In FTLD-tau vs αSyn, we observed a 2.80× fold change for p-tau212, 1.64× for p-tau181, 2.53× for p-tau212/tau368, and 1.16× for p-tau181/tau368. In FTLD-tau vs healthy controls, we observed a 2.46× fold change for p-tau212, 1.50× for p-tau181, 2.67× for p-tau212/tau368, and 1.67× for p-tau181/tau368.

Next, ROC analysis tested diagnostic accuracy in the ADNC-negative subcohort. Biomarkers in this subcohort showed a high AUC to discriminate FTLD-tau from FTLD-TDP: p-tau212 and p-tau181 reached AUC = 0.94 (95% CI 0.86–0.99) and AUC = 0.80 (95% CI 0.63–0.94), respectively. When tau368 ratio was applied, we observed that p-tau212/tau368 reached AUC = 0.95 (95% CI 0.87–1.00) and p-tau181/tau368 reached AUC = 0.93 (95% CI 0.81–1.00). Interestingly, we observed that p-tau212/t-tau ratio had a positive impact on the diagnostic accuracy when compared with p-tau212 alone. On the contrary, p-tau181/t-tau ratio had negative impact on the biomarker performance. (Supplementary Fig. 3b, Supplementary Table 4).

To discriminate FTLD-tau from all other groups (i.e., FTLD-TDP, αSyn, HC), we observed greater improvement of the accuracy when p-tau181/tau368 ratio was applied AUC = 0.88 (95% CI 0.80–0.95) vs p-tau181 alone AUC = 0.77 (95% CI 0.63–0.90), with only modest improvement for p-tau212/tau368 AUC = 0.87 (95% CI 0.79–0.94) compared to p-tau212 AUC = 0.84 (95% CI 0.74–0.92). (Supplementary Fig. 8; Supplementary Table 5). Stratifying FTLD-tau from not (FTLD-TDP, αSyn, Normal), DeLong’s test for two ROC curves shows significantly higher AUC for p-tau181/tau368 (0.88) than p-tau181/t-tau (0.58; *Z* = 3.43, *p* = 0.00061), but was not significant between p-tau212/tau368 (0.87) and p-tau212/t-tau (0.8; *Z* = 1.93, *p* = 0.054). Stratifying FTLD-tau from FTLD-TDP, DeLong’s test for two ROC curves shows significantly higher AUC for p-tau181/tau368 (0.93) than p-tau181/t-tau (0.72; *Z* = 2.34, *p* = 0.019), but no difference between p-tau212/tau368 (0.95) and p-tau212/t-tau (0.96; *Z* = − 0.17, *p* = 0.87). (Supplementary Fig. 9). Together, these results show that p-tau biomarkers alone are increased in FTLD-tau in comparison to FTLD-TDP, and they have significant diagnostic accuracy to discriminate between types and even from other neurodegenerative diseases, when amyloid pathology is excluded. Median fold-change and diagnostic accuracy for p-tau181 are further increased when intra-individual correction for tau368 is applied by using a ratio, with significantly better performance over p-tau181/t-tau ratio.

### Levels of CSF p-tau biomarkers with tau368 ratio are higher in most FTLD-tau subtypes than in FTLD-TDP subtypes

Due to the neuropathological heterogeneity within FTLD-tau and FTLD-TDP groups, we also performed sensitivity analyses to compare subtypes of each group; we observed that mean-fold changes in p-tau/tau368 ratios in FTLD-tau compared to FTLD-TDP were greater for all the diseases but PSP (Supplementary Fig. 10). Together these results show that p-tau biomarkers alone are increased in specific diseases classified as FTLD-tau in comparison to specific FTLD-TDP types; however, a special consideration might need to be taken regarding PSP.

### CSF tau biomarkers are correlated with brain tau in the ADNC-negative subcohort

**Fig. 4:**
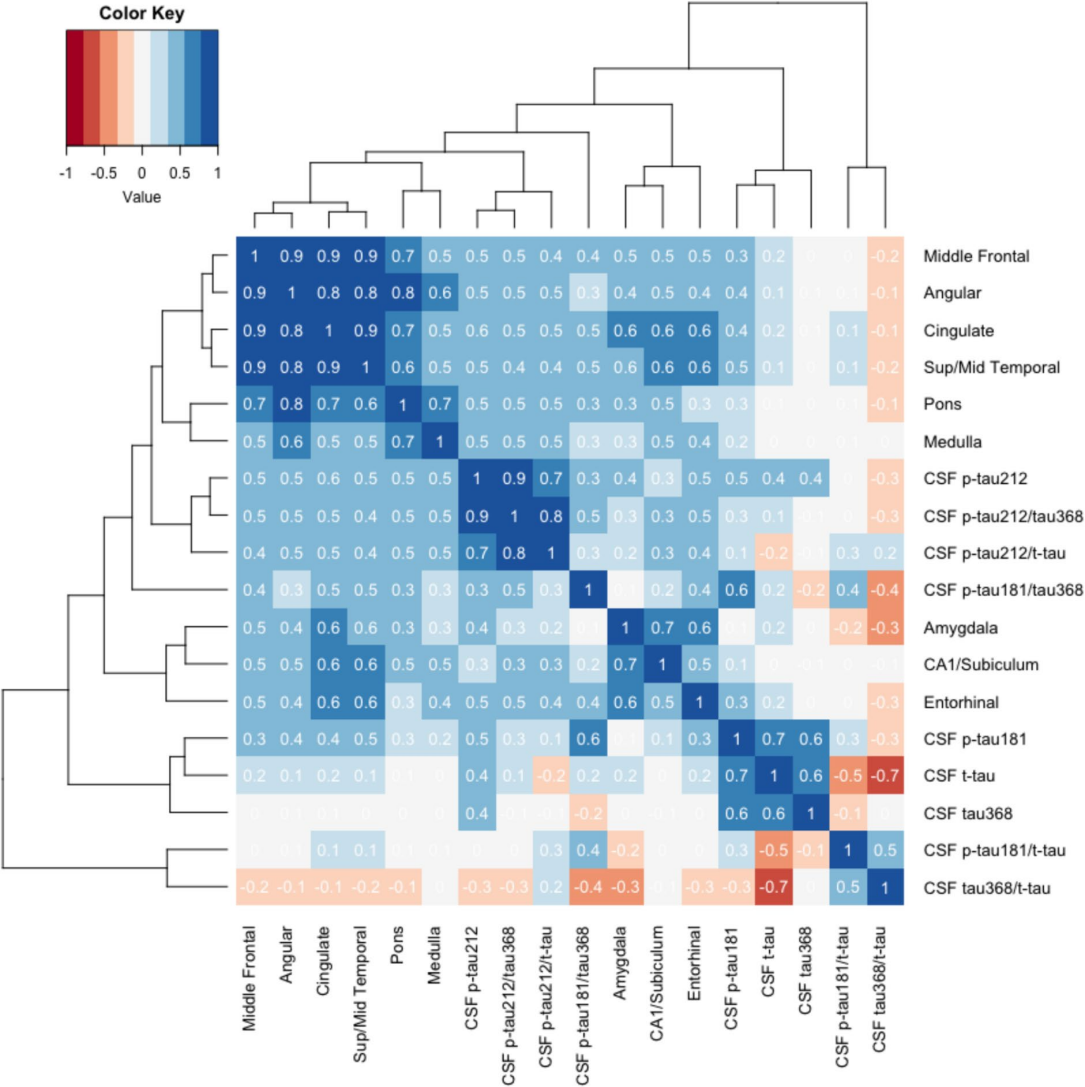
Correlations with regional brain tau burden in ADNC-negative subcohort. Spearman correlations tested associations between tau biomarkers and tau burden across the brain regions. Spearman’s rho is reported. White cells indicate *p* > 0.05

Then, we evaluated associations of tau biomarkers with core AD biomarkers in the ADNC-negative subcohort. CSF p-tau181, p-tau212, tau368, and CSF t-tau were statistically significantly correlated with each other, but not with the CSF Aβ42/Aβ40 ratio, Thal Phase, or brain Aβ (Supplementary Figs. 11, 12). Similarly, to the whole cohort (i.e., including primary ADNC and FTLD/αSyn with secondary ADNC), in the ADNC-negative subcohort we observed significant correlations between average brain tau burden for p-tau212, p-tau212/tau368, p-tau181/tau368, p-tau212/t-tau (Supplementary Fig. 13) *r* = (0.33–0.55, all *p* < 0.02). We did not observe a significant correlation for p-tau181, tau368, t-tau, p-tau181/t-tau and tau368/t-tau (all *p* ≥ 0.89). p-Tau biomarkers and their respective ratios with tau368 were significantly correlated with most of the brain regions. Interestingly, we do not observe significant correlation of p-tau181 and p-tau181/tau368 ratio with tau burden in the amygdala region (Fig. 4). This result further confirms that CSF Aβ42/Aβ40 ratio reflects amyloid pathology in the brain, since no correlations were observed when there is no amyloid pathology. Those results also show that the increase of p-tau in FTLD-tau is independent of the amyloid levels in the brain.

### Tau368 improves the discriminative effect of p-tau biomarkers in clinical variants of FTLD in the ADNC-negative subcohort

**Fig. 5:**
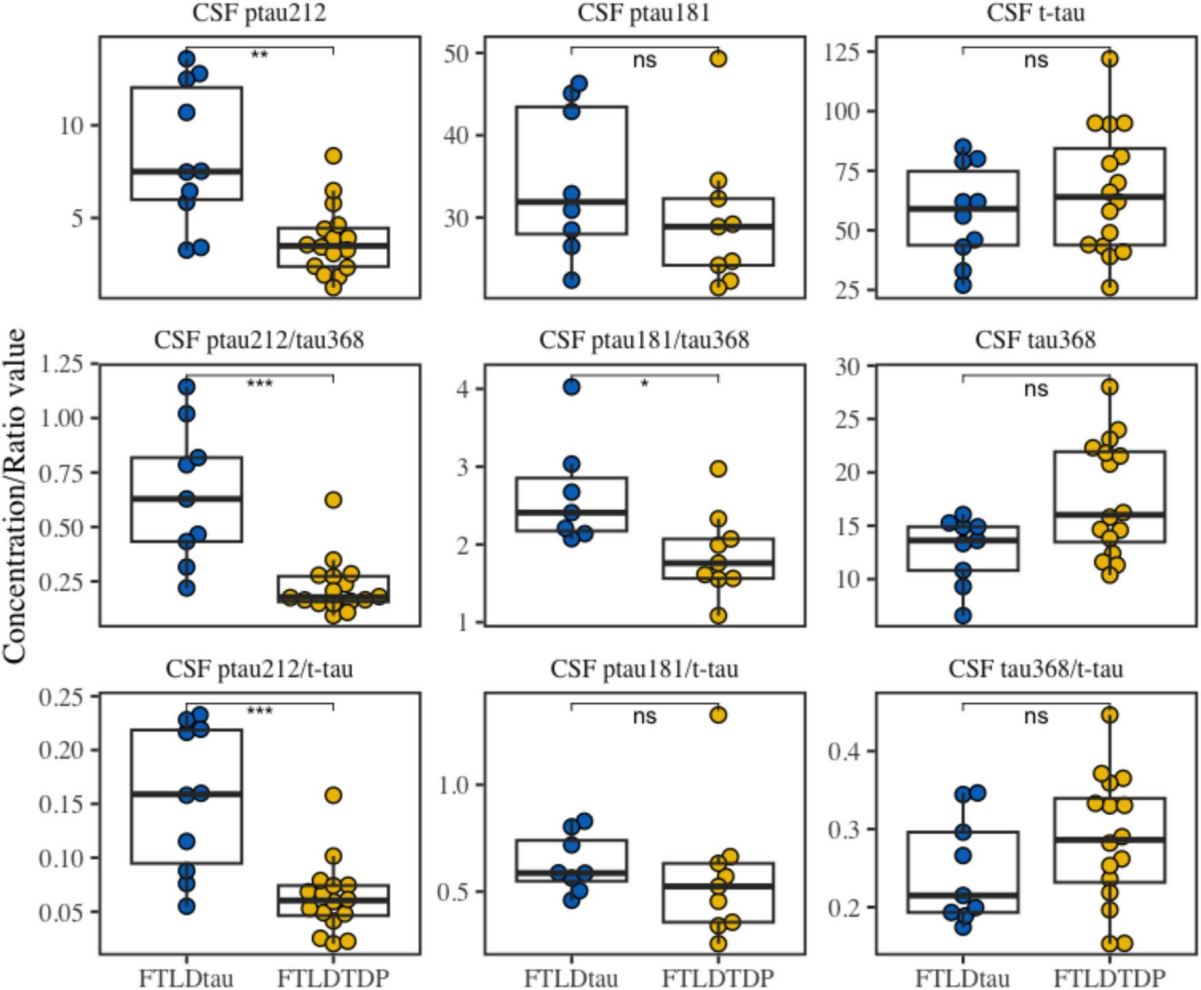
Levels of biomarkers in behavioral variants of FTLD. The figure shows levels of tau biomarkers in clinically characterized behavioral variants of FTLD (bvFTD-tau *n* = 8; bvFTD-TDP *n* = 12) in ADNC-negative participants from FTLD-tau and FTLD-TDP groups

Finally, we have evaluated whether levels of biomarkers are different in clinical variants of FTLD. P-tau212 was significantly increased in participants presenting bvFTD with underlying tau pathology, compared to those with underlying TDP-43 pathology (*p* < 0.01). For p-tau181, tau368, t-tau we did not see any statistically significant difference (Fig. 4). However, when the ratio with tau368 was applied, we observed that p-tau181/tau368, the discrimination between bvFTD-tau and bvFTD-TDP became statistically significant (*p* < 0.001). On the contrary, the p-tau181/t-tau ratio did not significantly improve the discrimination between groups (Fig. 5). Similar results were observed when people with PPA were included into the statistical analysis, favoring the use of tau368 instead of t-tau to differentiate clinical variants of FTLD (Supplementary Fig. 14). This improvement in discrimination shows the striking impact of using tau368 ratio as an inter-individual “controller” to help differentiate underlying proteinopathies in clinical variants of FTLD.

### Tau368 mediates the correlation of p-tau biomarkers in the FTLD-tau group

Finally, we confirm that tau368 is negatively associated with tau pathology in brains of FTLD group and mediates the correlation of p-tau biomarkers. To compare magnitude of correlations, we perform Pearson’s r correlations. p-tau212, p-tau181, and t-tau were not correlated with tangle pathology in FTLD-tau group (all *p* ≥ 0.26). CSF tau368 was negatively correlated with tau burden, although this did not reach significance (*r* = − 0.44, 95% CI − 0.75–0.01, *p* = 0.057) (Supplementary Fig. 15). However, after normalizing p-tau and t-tau biomarkers for tau368 we observe significant correlations for p-tau212/tau368 ratio (*r* = 0.53, 95% CI 0.08–0.8, *p* = 0.024), p-tau181/tau368 (*r* = 0.5, 95% CI 0.04–0.78, *p* = 0.036), and tau368/t-tau ratio (*r* = − 0.58, 95% CI − 0.82 to − 0.17, *p* = 0.0093). Hittner’s *Z* showed that p-tau212/tau368 and p-tau181/tau368 had significantly stronger correlations with tau pathology than p-tau212/t-tau (*r* = 0.07, 95% CI − 0.39–0.49, *p* = 0.78; *Z* = 2.61, *p* = 0.009) and p-tau181/t-tau (*r* = − 0.27, 95% CI − 0.64–0.19, *p* = 0.24; *Z* = 2.61, *p* = 0.009). Those results confirm the dual functionality of the tau368 immunoassay as a tau pathology-related inter-individual “controller” that outperforms t-tau.

## Discussion

In this study, we demonstrate that including tau368 as a ratio improves the sensitivity of p-tau biomarkers to differentiate autopsy-verified FTLD-tau from FTLD-TDP in no/low ADNC groups. Increased levels of p-tau/tau368 ratio are observed in antemortem CSF taken from participants presenting behavioral and language variants resulting from FTLD-tau, when compared to participants presenting the same clinical manifestations but with underlying TDP-43 pathology. To ensure the reliability of those findings, we performed a wide range of confirmatory experiments and analyses. Specifically, we confirmed the presence of tau368 fragments in the hippocampal and frontal cortex in a large spectrum of neurodegenerative diseases, including AD, αSyn, and FTLD. We examined antemortem levels of tau species in CSF of postmortem confirmed patients with a range of neurodegenerative ADRD. We first analyzed data in the whole cohort (healthy controls, AD, FTLD-tau, FTLD-TDP, αSyn, αSyn + AD) and then in the ADNC-negative subcohort (no/low ADNC; negative CSF Aβ42/Aβ40). Those experiments additionally address the complex pathophysiology of CSF tau fragments, confirming (1) dual roles of t-tau as an amyloid-driven biomarker in AD, but also a biomarker of neurodegeneration in FTLD; (2) that the greatest increase of p-tau levels in CSF is in AD; however, it is also observed in FTLD-tau, (3) the role of tau368 fragment as a tangle-related inter-individual variability controller for p-tau biomarkers.

CSF p-tau and t-tau biomarkers have established utility to diagnose AD [2, 5, 66], whereas data regarding tau368 is limited. While the use of the tau368 with t-tau has been proven to accurately discriminate biomarker-positive AD from biomarker-negative controls and to correlate inversely with severity of tau pathology as measured by tau PET [10, 85, 113], this is to our knowledge the first study to evaluate the p-tau/tau368 ratio. We confirmed that CSF p-tau181, p-tau212 levels, and tau368/t-tau ratio are efficient biomarkers to discriminate AD from other neurodegenerative diseases and healthy controls. Further, we confirm that antemortem levels of p-tau212 and p-tau181 are increased in autopsy-confirmed ADNC, with the highest median fold change observed for p-tau212. Concordantly with previous data, we confirm that tau368 alone did not reach a significant fold increase to discriminate AD [113], yet the tau368/t-tau ratio has acquired significant accuracy. An important and new finding is that both p-tau212/tau368 and p-tau181/tau368 ratios improved the accuracy of p-tau biomarkers. p-Tau/t-tau ratio worsened the accuracy of p-tau biomarkers. As expected, in the whole cohort, p-tau and p-tau/tau368 ratios were negatively correlated with the CSF Aβ42/40 ratio, and strongly positively correlated with Thal phases and Braak staging.

Together, those findings confirm the high increase of p-tau181, p-tau212, and t-tau in AD versus other neurodegenerative disorders or healthy controls, strengthening the high utility of these biomarkers. This increase is tightly correlated with the CSF Aβ42/Aβ40 and Thal phase, providing another proof for an amyloid-related increase of CSF p-tau or t-tau species in AD. Levels of tau368 were correlated with p-tau181, p-tau212, and t-tau, but not with CSF Aβ42/Aβ40 ratio, indicating shared biological mechanisms responsible for the presence of those tau species in CSF, and justifying the use of this fragment as an inter-individual variability controller. For ADNC, p-tau/tau368 ratio provided additional diagnostic value to biomarkers with established utility, suggesting possible improvement in their use in clinical trials for screening, diagnostic, or treatment efficacy monitoring purposes.

Next, we examined these tau biomarkers in the context of ADNC-negative primary tauopathies (i.e., FTLD-tau). In our ADNC-negative subcohort, we observed increased levels of both p-tau212 and p-tau181 in FTLD-tau compared to non-tauopathies including FTLD-TDP, αSyn, and healthy controls. The highest median fold change was observed for p-tau212/tau368 levels in FTLD-tau vs. FTLD-TDP. This confirms that p-tau levels are also increased in FTLD-tau. However, the changes are less prominent than in AD. Great discriminative power was observed for both p-tau212, which achieved AUC = 0.85 to differentiate between FTLD-tau and FTLD-TDP and p-tau181, which has AUC = 0.81, We observed improvement in AUC for p-tau212/tau368, reaching AUC = 0.86, and for p-tau181/tau368 reaching AUC = 0.84. Interestingly, p-tau212/t-tau improved the diagnostic accuracy, reaching AUC = 0.88, whereas p-tau181/t-tau worsened the accuracy, performing at AUC = 0.75. This suggests phosphorylation/fragment-specific patterns in FTLD, and favors using p-tau212/t-tau or p-tau212/tau368 ratios to discriminate FTLD-tau from FTLD-TDP. Similarly, to discriminate FTLD-tau from other participants in ADNC-negative subcohort, the greatest improvement was observed for p-tau181 (AUC = 0.77 vs. AUC = 0.83 for the p-tau181/tau368 ratio). This finding confirms that intra-individual variation might play a greater role in diagnostic accuracy for FTLD-tau, where the fold changes are not as high as for AD. Indeed, the histopathologic density of tau pathology in ADNC is much higher than in FTLD-tau and may influence CSF p-tau species [57]. Moreover, there are also structural differences in ADNC PHF compared to filaments in FTLD-tau [25], and greater relative white matter burden in FTLD-tau compared to ADNC [89] which could influence CSF tau biomarkers as well.

In the ADNC-negative subcohort, levels of tau368/t-tau levels were decreased in both FTLD-tau and FTLD-TDP in reference to healthy controls, a phenomenon that was previously observed for tau368/total tau ratio [29] and another MTBR peptide—MTBR-tau282 [48]. In the sensitivity analysis, the FTLD pathologic subgroups (Supplementary Fig. 10) were divided into specific neurodegenerative diseases; median fold levels of p-tau212 or p-tau212/tau368 ratio were higher in all FTLD-tau but PSP, compared to all FTLD-TDP. For the p-tau181/tau368 ratio, we observe that median fold levels in all the diseases, except PSP are higher for FTLD-tau compared to FTLD-TDP. This study is also another confirmation that although we observe an increase of fibrillar tau in the PSP, levels of the soluble tau remain distinct from other tauopathies. Previously published studies showed increased p-tau181 levels in CBS, in reference to PSP [1, 11, 17] additionally, levels of p-tau in PSP are often referred to be lower than in controls [43, 109]. PSP tauopathy may begin in subcortical structures and has varying inter-individual density of neuronal and glial tau inclusions in the cortex postmortem [72] which could influence p-tau measures here. However, there are also studies reporting no differences between biomarkers in those conditions. Indeed, in one study when PSP and CBD are included as FTLD-tau, the difference in CSF p-tau along with inflammatory and lysosomal markers was less robust compared to FTLD-TDP [20]. In the analyses that exclude PSP, we once again observe improved AUC for the ratios, reaching AUC = 0.95 for p-tau212/tau368 in discriminating FTLD-tau from FTLD-TDP and AUC = 0.88 for p-tau181/tau368 in discriminating FTLD-tau vs. other groups.

In our rare autopsy-confirmed dataset, we are limited in our ability to test sensitivity analyses within specific clinical subgroups; however, we observe increased levels of p-tau212, p-tau181, and representative tau368 ratios in PiD, CBD and *MAPT*, compared with FTLD types A and B, which indicated potential utility in discriminating heterogeneous syndromes such as bvFTD. Knowing that, we analyzed p-tau, t-tau and tau368 levels in participants presenting bvFTD. We observed that p-tau212 levels were increased in bvFTD with underlying tau pathology, compared to those with TDP-43 pathology. p-Tau181 alone was not significant. P-tau212 was previously shown to have better diagnostic performance than p-tau181 in discriminating between AD and control groups at different stages of the disease [65, 66]. This study is an example that specific p-tau biomarkers might have different utility in non-AD tauopathies. Although in this study we are unable to examine specific molecular mechanisms responsible for this distinct performance, we know that Thr212 is within the sequence that is targeted by more kinases than Thr181, and is susceptible to being phosphorylated in lower concentrations of the dual-specificity tyrosine phosphorylation-regulated kinase 1A (DYRK1A) than other p-tau biomarkers [64, 80]. In FTLD-tau, DYRK1A levels are increased [7, 26], providing a possible biological mechanism underlying the differences in p-tau abilities to discriminate FTLD-tau from FTLD-TDP. Still, the p-tau181/tau368 ratio showed statistically significant increase to discriminate between FTLD-tau and FTLD-TDP. This further supports that using tau368 as a proxy to normalize for the inter-individual variation can show significantly increased levels of p-tau and is an effective way to utilize p-tau biomarkers’ role to discriminate clinical variants of FTLD with underlying tau pathology in reference to TDP-43 pathology, even in a small number of participants per group.

We also observe increased levels of p-tau212, p-tau181, and representative tau368 ratios in PiD and GGT, compared with FTLD-TDP type C, suggesting possible utility to discriminate pathological substrate of svPPA and other temporal variants of FTD; however, there is not a large enough sample to make a proper statistical analysis to further support this observation and future work is needed in larger autopsy datasets to test clinical syndrome and pathological substrate interactions in FTD. However, when we pooled participants presenting with PPA with those presenting with bvFTD, once again we have found that tau368 ratio was a highly influential for diagnostic accuracy using p-tau181 levels. Tau368 also improved detection of tau pathology when combined with p-tau212. Despite differences in the levels of PSP when compared with other tauopathies, participants who had PSP and presented specific clinical variants were included in all those analyses.

Another finding from this study is that in the ADNC-negative subcohort we do not observe a correlation of p-tau, t-tau, or tau368 with the CSF Aβ42/Aβ40 and Thal phase, indicating that an increase of the biomarkers in FTLD-tau is independent of amyloid levels and providing another indication of amyloid-dependent increase of p-tau in AD CSF.

The next finding is that p-tau181, p-tau212, and respective p-tau/tau368 ratios were correlated with the brain tau burden in the ADNC-negative subcohort. On the contrary, tau368 or tau368/t-tau levels were not correlated with tau burden in the ADNC-negative subcohort. This finding is not surprising since the same results were observed in previous publications, where levels of tau368[29, 113], or other MTBR peptides such as MTBR-tau275, were found to not be altered in FTLD, and the ratio of these with t-tau was lower in both FTLD-TDP and FTLD-tau [29, 48]. Additionally, levels of CSF MTBR-tau275/t-tau and MTBR-tau282/t-tau were also observed to be significantly lower in CBD, compared to PSP, without any significant differences observed for the MTBR-tau275 or MTBR-tau282 peptides alone [48].

Relatively unaltered levels of tau368 in healthy controls and other neurodegenerative diseases are not surprising for us since it is well-established that soluble tau species present in CSF are different than insoluble tau species which we observe in the brain [6, 52, 58, 70, 104]. Additionally, tau368 immunohistochemistry showed nuclear and cytoplasmic presence of tau368 in the healthy controls, and additionally, filamentous structures in AD cases [10]. The analogical situation happens with the Aβ40 peptide, which forms the core of the plaque in sporadic AD brain [94], similarly to tau368 which forms the core in the tau aggregates in AD [28] and FTLD [33] however, the Aβ40 peptide levels are unaltered in the AD [42, 44, 112]. Alternatively, pathological secretion of the MTBR fragment might be counterbalanced by the increased aggregation, which would reduce levels of those biomarkers in CSF. A mechanism that may underlie these findings and could be evaluated in further research is the involvement of the lysosomal system. Out of 28 possible ubiquitination sites on tau protein, most of them are on the proline-rich region and MTBR, suggesting enhanced degradation of those fragments [123].

Having in mind the complex physiology of the tau368 fragment in CSF, we checked the correlation of tau368 with tau burden within the sole FTLD-tau group, aiming to receive a clear answer regarding tangle association. The correlation was significantly negative, possibly reflecting accumulation of this fragment in the brain and proving the association between tau368 and tangle pathology. Future work in larger cohorts will help confirm these trends and validate with pathology. Nonetheless, these results support the value of CSF p-tau normalized to MTBR fragments in FTLD-tau and that these levels reflect the intermediate tau burden observed postmortem compared to ADNC.

It is also important to mention that t-tau levels were also reported to be increased in FTLD-TDP in comparison to FTLD-tau [12]. Additionally, t-tau is increased in Creutzfeldt–Jakob disease (CJD) [22, 91, 116], and acute stroke [45], which are diseases that per se do not have a neuropathological accumulation of tau protein in the brain [52]. This might be explained because t-tau is a marker of neurodegeneration, rather than a marker of tau pathology [9]. On the other hand, studies confirm an increase of CSF t-tau in relation to the prominence of amyloid pathology in mice overexpressing human amyloid precursor protein (APP). Importantly, those mice do not develop tangles, providing a direct link to Aβ fragments [83]. That relation was also confirmed in human CSF [108]. Those studies are another confirmation that t-tau is a biomarker whose levels are driven by amyloid in AD, but also that is driven by neurodegeneration in other diseases, with very limited data regarding its secretion related to tangle pathology.

This is the first use of the p-tau/MTBR-tau ratio for the purpose of inter-individual “controller”, however, the concept has been described previously [44, 97]. Since the variation of the CSF protein levels is influenced by multiple factors including aging [88], CSF turnover in different neurodegenerative diseases [61], CSF hydrodynamics [62] or circadian variation of the CSF production [99], including a ‘controller’ protein can help to adjust these factors Additionally, individual, physiological CSF variability is an already established fact [69]. Recent studies measured CSF levels of brain-derived proteins, and alterations in proteins, such as Ig domain containing 1 protein (LINGO1), neuronal pentraxin 2 (NPTX2), prodynorphin (PDYN), cholecystokinin (CCK), and neuronal pentraxin receptor (NPTXR), were observed as well as slightly elevated levels of proteins in AD, ALS, and PPA, independently of the disease pathology. Authors next adjusted for median CSF protein levels which provided a better association with the disease [97]. Very importantly, CSF p-tau and t-tau levels are suggested to be affected by inter-individual variability [69, 97]. On top of that, levels of p-tau and t-tau were proven to be vulnerable to pre-analytical handling, for example, collection tubes [77]. Here, we use standardized operating procedures in CSF collection and storage to minimize preanalytical sources of variation. Although normalization of CSF p-tau181 with Aβ40 had beneficial effects [41], p-tau, t-tau, and tau368 originate from the same protein and are correlated in the CSF, strengthening the strong biological link between those analytes. Therefore, we propose to use tau368 as an optimal reference protein to “control” for the inter-individual variation for the p-tau measurements in the CSF.

To summarize, in this unique autopsy cohort, we were able to find accurate diagnostic biomarkers to differentiate between diseases with strong clinical overlap. Using the approach that allowed us to normalize the inter-individual variation, we were able to increase the diagnostic accuracy of our immunoassays. Our research also provides a valuable replication of previous findings, especially regarding the levels of the biomarkers in autopsy-confirmed neurodegenerative diseases and healthy controls. Another strong point of this study is the possibility of used immunoassays to be implemented globally.

Our study has also a few limitations. Some diseases (e.g., multiple system atrophy) are missing in our analysis; therefore, the observed diagnostic performances may not be generalizable to neurology clinics. Second, this study included extremely rare autopsy data from patients with antemortem CSF, necessary to test biomarkers in sporadic FTLD which lacks diagnostic biomarkers. Still, sample sizes were relatively small and likewise clinical, racial, and pathological diversity were limited. Thus, to ensure generalizability of our findings, future replication is necessary in larger cohorts that include sporadic bvFTD, different pathological subtypes, and socio-demographic diversity. Third, disease groups were symptomatic at time of CSF collection, with average disease onset of 4.4 years. Future longitudinal studies are also needed to test whether biomarkers are dynamic or stable over disease course, and to establish whether biomarkers are accurate early in disease course. Here we focused on CSF, and future work can compare biomarkers and dynamics in plasma. Thus, we emphasize this is important initial validation work, but replication and extension will be required before these assays can be applied for widespread clinical use. tau368 staining for FTLD-tau is ongoing and more quantitative and qualitative neuropathological analyses to address heterogeneity and disease-stage dependency will be addressed in future. In this study, PHF-1 antibody was used to quantify Average brain tau metric or brain tau burden.

## Conclusions

We confirm that antemortem CSF p-tau levels correctly identify autopsy-verified AD, and they correlate with tau pathology in the brain in both AD and FTLD-tau. Moreover, in such a great exigency to find biomarkers accurately identifying other dementia forms in this proof-of-concept study, we validate that after excluding Aβ pathology, we can differentiate FTLD-tau from FTLD-TDP using p-tau biomarkers. We can further increase the diagnostic accuracy with the use of tangle-related inter-individual “controller”—tau368. That role was the most prominent in the differentiation between bvFTLD and PPA-FTLD variants. This study will support potential improvement in diagnosis and differentiation of the FTLD spectrum, allowing us to differentiate diseases with overlapping behavioral or semantic manifestations. Accurate diagnosis will support recruitment for clinical trials and will allow patients to receive appropriate therapy. By increasing specificity and sensitivity of the used immunoassays, our research will improve the diagnosis due to reduced false positive or false negative findings.

## Supplementary Information

Below is the link to the electronic supplementary material.

Supplementary file1 (DOCX 5556 KB)

## Data Availability

Blinded anonymized data are available on reasonable request from the corresponding author. The request will be reviewed by the investigators and respective institutions to verify if data transfer is in agreement with EU legislation on general data protection or is subject to any intellectual property or confidentiality obligations.

## Code availability

The codes used for the data analyses in this study can be requested from the corresponding author.
