# Supplementary material for: Tau368 improves p-tau diagnostic accuracy for FTLD-tau from FTLD-TDP
Source: Acta Neuropathol. 2026 Jun 24;151(1):71. doi: 10.1007/s00401-026-03042-1 (PMC13294230; doi:10.1007/s00401-026-03042-1)
Supplement: Supplementary file 1 — Supplementary file1 (DOCX 5556 KB) [file 401_2026_3042_MOESM1_ESM.docx]

**Supplementary Table 1. Demographic characteristics of the brain samples from UCSD**

| **AGE at visit** | **SEX** | **APOE ε4** | **MMSE** | **BRAAK** | **THAL** | **Pathology Diagnosis** |
| --- | --- | --- | --- | --- | --- | --- |
| 92 | F | 33 | 27 | 5 | 4 | Alzheimer’s disease |
| 83 | F | 33 | 14 | 4 | NA | Alzheimer's disease; Diffuse Lewy body disease |
| 93 | M | 33 | 25 | 1 | 0 | Lewy body disease- limbic (transitional) type; Microinfarcts, old |
| 59 | F | 33 | 9 | 1 | 1 | Frontotemporal dementia; Hippocampal sclerosis |
| 71 | F | 44 | 13 | 6 | 5 | Alzheimer's disease; Amyloid angiopathy; Lewy body pathology, limbic (transitional) type; TDP-43 proteinopathy |
| 70 | F | 34 | 22 | 2 | NA | Diffuse Lewy body disease; Alzheimer's changes |
| 76 | M | 33 | 16 | 6 | 5 | Alzheimer's disease; Amyloid angiopathy; Lewy body pathology, neocortical (diffuse) type |
| 89 | M | 33 | 23 | 2 | 5 | Lewy body disease- brain stem predominant type; Alzheimer's changes; Microinfarcts, old. |
| 83 | M | 34 | 30 | 6 | 5 | Alzheimer’s disease |
| 81 | M | 44 | 26 | 4 | 5 | Lewy body disease- neocortical (diffuse) type; Alzheimer's disease |
| 77 | M | 34 | 12 | 3 | 4 | Lewy body pathology, neocortical (diffuse); Alzheimer's disease; TDP-43 proteinopathy |
| 83 | F | 33 | 25 | 3 | 4 | Alzheimer's disease; Lewy body disease- neocortical (diffuse) type |
| 70 | M | 34 | 24 | 3 | 4 | Alzheimer's disease; Lewy body disease- neocortical (diffuse) type |
| 71 | M | 34 | 12 | 5 | 5 | Alzheimer’s disease; Amyloid angiopathy; Lewy body pathology, amygdala predominant type; TDP-43 proteinopathy |
| 89 | F | 33 | 26 | 2 | 5 | Alzheimer’s changes |
| 79 | M | 23 | 29 | 2 | 0 | Normal Control |

Abbreviations: Apolipoprotein E (APOE); Tau classification according to Braak staging (BRAAK)
mini-mental state examination(MMSE); TAR-DNA binding protein 43 (TDP-43)

**Supplementary Table 2. Demographic characteristics of the amyloid-negative biomarker subcohort**

|  | FTLDtau | FTLDTDP | αSyn | Normal | p | Missing |
| --- | --- | --- | --- | --- | --- | --- |
| n | 24 | 29 | 12 | 41 |  |  |
| Age at Onset (years) | 65.0 [59.0, 70.0] | 60.0 [55.0, 64.5] | 61.5 [57.0, 66.0] | -- | 0.152 | 42 |
| Age at CSF (years) | 69.5 [62.5, 72.0] | 63.0 [58.0, 68.0] | 71.0 [67.2, 78.2] | 66.0 [61.0, 70.0] | 0.019 | -- |
| Age at Death (years) | 72.0 [66.0, 76.5] | 66.0 [60.5, 73.0] | 78.5 [73.2, 81.0] | 76.0 [76.0, 76.0] | 0.014 | 43 |
| CSF to Death (years) | 4.0 [2.0, 5.5] | 4.0 [2.5, 5.0] | 4.5 [1.8, 6.2] | 6.0 [6.0, 6.0] | 0.711 | 43 |
| Sex = Male (%) | 13 (54.2%) | 16 (55.2%) | 11 (91.7%) | 13 (31.7%) | 0.003 | -- |
| Self-reported Race (%) |  |  |  |  | 0.045 | -- |
| Asian | 0 | 1 (3.4%) | 0 | 0 |  |  |
| Black or African American | 0 | 1 (3.4%) | 0 | 9 (22.0%) |  |  |
| More than one Race | 0 | 1 (3.4%) | 0 | 2 (4.9%) |  |  |
| White | 24 (100.0%) | 26 (89.7%) | 12 (100.0%) | 30 (73.2%) |  |  |
| ClinicalDx (%) |  |  |  |  | <0.001 | -- |
| Normal | 0 | 0 | 0 | 41 (100.0%) |  |  |
| Amnestic | 2 (8.3%) | 0 | 1 (8.3%) | 0 |  |  |
| CBS | 5 (20.8%) | 1 (3.4%) | 0 | 0 |  |  |
| lvPPA | 2 (8.3%) | 0 | 0 | 0 |  |  |
| naPPA | 2 (8.3%) | 2 (6.9%) | 0 | 0 |  |  |
| svPPA | 0 | 1 (3.4%) | 0 | 0 |  |  |
| bvFTD | 10 (41.7%) | 16 (55.2%) | 0 | 0 |  |  |
| ALS | 0 | 5 (17.2%) | 0 | 0 |  |  |
| PSP | 3 (12.5%) | 0 | 0 | 0 |  |  |
| PD/PDD | 0 | 0 | 10 (83.3%) | 0 |  |  |
| DLB | 0 | 0 | 1 (8.3%) | 0 |  |  |
| Vascular | 0 | 1 (3.4%) | 0 | 0 |  |  |
| Dementia NOS | 0 | 3 (10.3%) | 0 | 0 |  |  |
| Mutation (%) |  |  |  |  | <0.001 | -- |
| None | 18 (75.0%) | 13 (44.8%) | 12 (100.0%) | 41 (100.0%) |  |  |
| C9orf72 | 0 | 10 (34.5%) | 0 | 0 |  |  |
| GRN | 0 | 5 (17.2%) | 0 | 0 |  |  |
| MAPT | 6 (25.0%) | 0 | 0 | 0 |  |  |
| TARDBP | 0 | 1 (3.4%) | 0 | 0 |  |  |
| ADNC = Low (%) | 15 (71.4%) | 10 (50.0%) | 9 (75.0%) | -- | -- | 53 |
| Thal Phase (%) |  |  |  |  | -- | 52 |
| 0 | 6 (27.3%) | 10 (50.0%) | 3 (25.0%) | -- |  |  |
| 1 | 12 (54.5%) | 7 (35.0%) | 5 (41.7%) | -- |  |  |
| 2 | 4 (18.2%) | 3 (15.0%) | 4 (33.3%) | -- |  |  |
| Brain tau burden | 2.0 [1.8, 2.4] | 0.5 [0.2, 1.0] | 0.7 [0.6, 1.1] | -- | <0.001 | 55 |
| CSF p-tau181 | 40.5 [30.8, 47.1] | 28.9 [22.9, 32.3] | 25.8 [21.9, 31.6] | 28.2 [23.1, 32.8] | 0.004 | 18 |
| CSF t-tau | 56.0 [45.8, 79.2] | 58.0 [44.0, 83.0] | 36.0 [26.5, 49.0] | 44.0 [37.0, 57.7] | 0.001 | -- |
| CSF p-tau212 | 7.6 [5.1, 12.9] | 3.6 [2.1, 4.8] | 3.8 [3.0, 4.6] | 4.4 [3.1, 7.0] | <0.001 | 2 |
| CSF tau368 | 15.7 [13.4, 20.3] | 16.2 [13.8, 21.5] | 13.8 [10.3, 17.7] | 17.6 [14.8, 19.8] | 0.441 | 4 |
| CSF p-tau212/tau368 | 0.6 [0.3, 0.8] | 0.2 [0.2, 0.3] | 0.3 [0.2, 0.4] | 0.3 [0.2, 0.4] | <0.001 | 6 |
| CSF p-tau181/tau368 | 2.5 [2.1, 2.8] | 1.8 [1.6, 1.9] | 2.1 [1.3, 2.5] | 1.5 [1.3, 2.0] | <0.001 | 22 |
| CSF Aβ42/Aβ40 | 0.091 [0.081, 0.095] | 0.090 [0.078, 0.097] | 0.094 [0.088, 0.100] | 0.098 [0.094, 0.103] | 0.006 | 18 |
| CSF tau368/t-tau | 0.287 [0.216, 0.366] | 0.285 [0.236, 0.330] | 0.381 [0.314, 0.581] | 0.378 [0.327, 0.466] | <0.001 | 4 |
| CSF p-tau212/t-tau | 0.160 [0.097, 0.218] | 0.061 [0.043, 0.076] | 0.117 [0.089, 0.159] | 0.104 [0.084, 0.135] | <0.001 | 2 |
| CSF p-tau181/t-tau | 0.605 [0.568, 0.810] | 0.468 [0.377, 0.601] | 0.708 [0.592, 0.971] | 0.614 [0.532, 0.680] | 0.002 | 18 |

Abbreviations: Amyloid-β (Aβ); Amyotrophic Lateral Sclerosis (ALS); Alzheimer’s Disease (AD); Alzheimer’s Disease neuropathologic change (ADNC); Behavioural variant of Frontotemporal lobar degeneration (bvFTD); Cerebrospinal Fluid (CSF); chromosome 9 open reading frame 72 (C9orf72); clinically healthy individuals without cognitive impairment (normal); Corticobasal Syndrome (CBS); Frontotemporal Lobar Degeneration type tau (FTLD-Tau), Frontotemporal Lobar Degeneration type TDP (FTLD-TDP): Granulin (GRN); Lewy body disease (LBD); Lewy body disease with alpha-synuclein/Parkinson’s Disease (NSD; αSyn) and Lewy body disease with alpha-synuclein/Parkinson’s Disease with concominant AD co-pathology (NSD+AD; αSyn+AD); Microtubule-associated protein Tau (MAPT); Mild-Cognitive Impairment (MCI); Parkinson’s Disease (PD); Phosphorylated-tauX (p-tauX); Presenilin1 (PSEN); Primary Progressive Aphasia (PPA); Progressive Supranuclear Palsy (PSP); total-tau (t-tau).

**Supplementary Table 3. Receiver operating characteristic curves (ROC) to discriminate Alzheimer’s Disease (AD) from other participants.**

| AD vs. Not | AUC | AUC 95% CI | Threshold | Threshold 95% CI | Sensitivity | Specificity | Accuracy |
| --- | --- | --- | --- | --- | --- | --- | --- |
| ptau212/tau368 | 0.94 | 0.90 – 0.97 | 0.95 | 0.65 – 1.16 | 0.81 | 0.90 | 0.84 |
| ptau181/tau368 | 0.94 | 0.89 – 0.99 | 3.15 | 2.82 – 3.40 | 0.90 | 0.93 | 0.91 |
| ptau212 | 0.92 | 0.87 – 0.96 | 16.29 | 11.37 – 21.37 | 0.80 | 0.90 | 0.84 |
| ptau181 | 0.91 | 0.85 – 0.97 | 54.72 | 46.18 – 61.33 | 0.82 | 0.92 | 0.87 |
| p-tau212/t-tau | 0.89 | 0.83 – 0.95 | 0.22 | 0.18 – 0.25 | 0.81 | 0.88 | 0.84 |
| p-tau181/t-tau | 0.83 | 0.74 – 0.92 | 0.75 | 0.68 – 0.85 | 0.84 | 0.75 | 0.80 |
| tau368/t-tau | 0.79 | 0.70 – 0.87 | 0.26 | 0.21 – 0.30 | 0.74 | 0.66 | 0.70 |
| t-tau | 0.78 | 0.70 – 0.85 | 66.93 | 58.06 – 88.94 | 0.71 | 0.70 | 0.71 |
| tau368 | 0.60 | 0.48 – 0.70 | 18.58 | 14.36 – 23.87 | 0.40 | 0.64 | 0.50 |

**Supplementary Table 4. Areas under the curve to discriminate FTLD-tau from FTLD-TDP in amyloid-negative subcohort.** A. FTLD-tau excluding PSP (n=102), and B. FTLD-tau including PSP (n=106).

| A.  FTLD-Tau vs. FTLD-TDP | AUC | AUC 95% CI | Threshold | Threshold 95% CI | Sensitivity | Specificity | Accuracy |
| --- | --- | --- | --- | --- | --- | --- | --- |
| p-tau212/t-tau | 0.88 | 0.76 – 0.97 | 0.11 | 0.08 – 0.15 | 0.76 | 0.90 | 0.83 |
| p-tau212/tau368 | 0.86 | 0.72 – 0.96 | 0.36 | 0.24 – 0.54 | 0.74 | 0.85 | 0.79 |
| p-tau212 | 0.85 | 0.71 – 0.95 | 6.13 | 4.59 – 8.86 | 0.76 | 0.85 | 0.80 |
| p-tau181/tau368 | 0.84 | 0.68 – 0.97 | 2.06 | 1.93 – 2.28 | 0.78 | 0.82 | 0.80 |
| p-tau181 | 0.81 | 0.66 – 0.94 | 32.21 | 28.05 – 37.18 | 0.70 | 0.76 | 0.73 |
| p-tau181/t-tau | 0.75 | 0.57 – 0.90 | 0.57 | 0.49 – 0.68 | 0.70 | 0.65 | 0.68 |
| tau368 | 0.53 | 0.35 – 0.72 | 15.21 | 12.52 – 18.93 | 0.60 | 0.55 | 0.58 |
| t-tau | 0.51 | 0.33 – 0.68 | 75.85 | 50.96 – 106.92 | 0.68 | 0.35 | 0.52 |
| tau368/t-tau | 0.50 | 0.32 – 0.68 | 0.33 | 0.21 – 0.42 | 0.35 | 0.60 | 0.48 |

| B.  FTLD-Tau vs. FTLD-TDP  (Including PSP) | AUC | AUC 95% CI | Threshold | Threshold 95% CI | Sensitivity | Specificity | Accuracy |
| --- | --- | --- | --- | --- | --- | --- | --- |
| p-tau212/t-tau | 0.96 | 0.89 – 1.00 | 0.11 | 0.08 – 0.15 | 0.88 | 0.90 | 0.89 |
| p-tau212/tau368 | 0.95 | 0.87 – 1.00 | 0.37 | 0.26 – 0.54 | 0.88 | 0.85 | 0.86 |
| p-tau212 | 0.94 | 0.86 – 0.99 | 5.94 | 4.63 – 8.65 | 0.88 | 0.85 | 0.86 |
| p-tau181/tau368 | 0.93 | 0.81 – 1.00 | 2.06 | 1.92 – 2.30 | 0.87 | 0.82 | 0.84 |
| p-tau181 | 0.80 | 0.63 – 0.94 | 34.10 | 28.79 – 39.13 | 0.62 | 0.82 | 0.73 |
| p-tau181/t-tau | 0.72 | 0.54 – 0.88 | 0.57 | 0.46 – 0.73 | 0.62 | 0.65 | 0.64 |
| tau368/t-tau | 0.56 | 0.37 – 0.75 | 0.28 | 0.22 – 0.35 | 0.53 | 0.55 | 0.54 |
| tau368 | 0.50 | 0.31 – 0.69 | 14.97 | 11.70 – 18.92 | 0.65 | 0.55 | 0.59 |
| t-tau | 0.48 | 0.30 – 0.67 | 75.26 | 44.20 – 110.12 | 0.61 | 0.35 | 0.47 |

**Supplementary Table 5. Areas under the curve to discriminate FTLD-tau from other participants in amyloid-negative subcohort.** A. FTLD-tau excluding PSP (n=102), and B. FTLD-tau including PSP (n=106).

| A.  FTLD-Tau vs. Not | AUC | AUC 95% CI | Threshold | Threshold 95% CI | Sensitivity | Specificity | Accuracy |
| --- | --- | --- | --- | --- | --- | --- | --- |
| p-tau181/tau368 | 0.88 | 0.80 – 0.95 | 2.12 | 1.95 – 2.35 | 0.87 | 0.74 | 0.77 |
| p-tau212/tau368 | 0.87 | 0.79 – 0.94 | 0.42 | 0.28 – 0.58 | 0.78 | 0.81 | 0.80 |
| p-tau212 | 0.84 | 0.74 – 0.92 | 6.41 | 4.63 – 9.36 | 0.74 | 0.75 | 0.75 |
| p-tau212/t-tau | 0.80 | 0.68 – 0.90 | 0.13 | 0.10 – 0.16 | 0.68 | 0.79 | 0.77 |
| p-tau181 | 0.77 | 0.63 – 0.90 | 36.32 | 30.80 – 42.17 | 0.62 | 0.84 | 0.80 |
| tau368/t-tau | 0.70 | 0.55 – 0.84 | 0.28 | 0.23 – 0.33 | 0.53 | 0.75 | 0.71 |
| t-tau | 0.63 | 0.49 – 0.76 | 55.96 | 45.13 – 72.85 | 0.65 | 0.62 | 0.63 |
| p-tau181/t-tau | 0.58 | 0.42 – 0.74 | 0.69 | 0.52 – 0.90 | 0.38 | 0.75 | 0.68 |
| tau368 | 0.55 | 0.40 – 0.70 | 14.85 | 10.42 – 19.35 | 0.42 | 0.62 | 0.59 |

| B.  FTLD-Tau vs. Not (Including PSP) | AUC | AUC 95% CI | Threshold | Threshold 95% CI | Sensitivity | Specificity | Accuracy |
| --- | --- | --- | --- | --- | --- | --- | --- |
| p-tau181/tau368 | 0.83 | 0.73 – 0.91 | 2.06 | 1.83 – 2.32 | 0.78 | 0.73 | 0.74 |
| p-tau212/tau368 | 0.78 | 0.64 – 0.89 | 0.44 | 0.30 – 0.60 | 0.62 | 0.84 | 0.79 |
| p-tau212 | 0.76 | 0.64 – 0.88 | 6.52 | 4.74 – 9.52 | 0.61 | 0.79 | 0.75 |
| p-tau181 | 0.76 | 0.64 – 0.88 | 33.53 | 28.81 – 39.34 | 0.65 | 0.78 | 0.75 |
| p-tau212/t-tau | 0.72 | 0.59 – 0.84 | 0.13 | 0.11 – 0.16 | 0.57 | 0.80 | 0.75 |
| tau368/t-tau | 0.66 | 0.52 – 0.79 | 0.29 | 0.23 – 0.37 | 0.55 | 0.70 | 0.67 |
| t-tau | 0.62 | 0.48 – 0.74 | 51.25 | 42.44 – 66.84 | 0.67 | 0.59 | 0.60 |
| p-tau181/t-tau | 0.61 | 0.47 – 0.74 | 0.64 | 0.54 – 0.78 | 0.45 | 0.63 | 0.59 |
| tau368 | 0.52 | 0.38 – 0.66 | 15.18 | 10.59 – 20.98 | 0.50 | 0.60 | 0.58 |


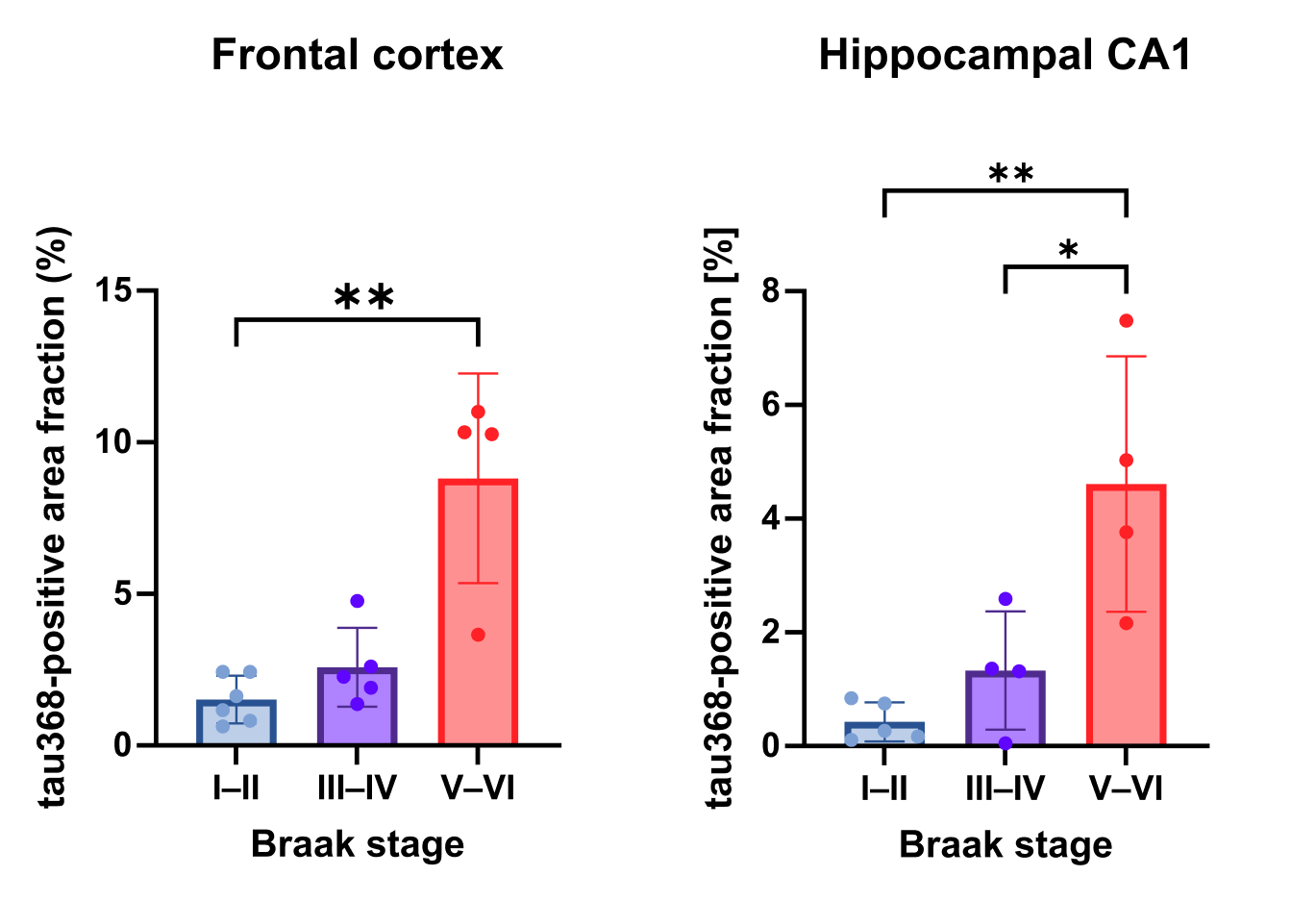


**Supplementary Figure 1. Quantification of tau368-positive area fraction across Braak stage groups in frontal cortex and hippocampal CA1.**

Tau368-positive area fraction was quantified in post-mortem brain sections from the frontal cortex and hippocampal CA1 and grouped by Braak stage as I–II, III–IV, and V–VI. Bars represent group mean ± SD, and dots represent individual cases. In the frontal cortex, tau368-positive area fraction was higher in Braak V–VI than in Braak I–II. In hippocampal CA1, tau368-positive area fraction was higher in Braak V–VI than in both Braak I–II and Braak III–IV. Statistical comparisons are indicated on the graph: * p < 0.05; ** p < 0.01.


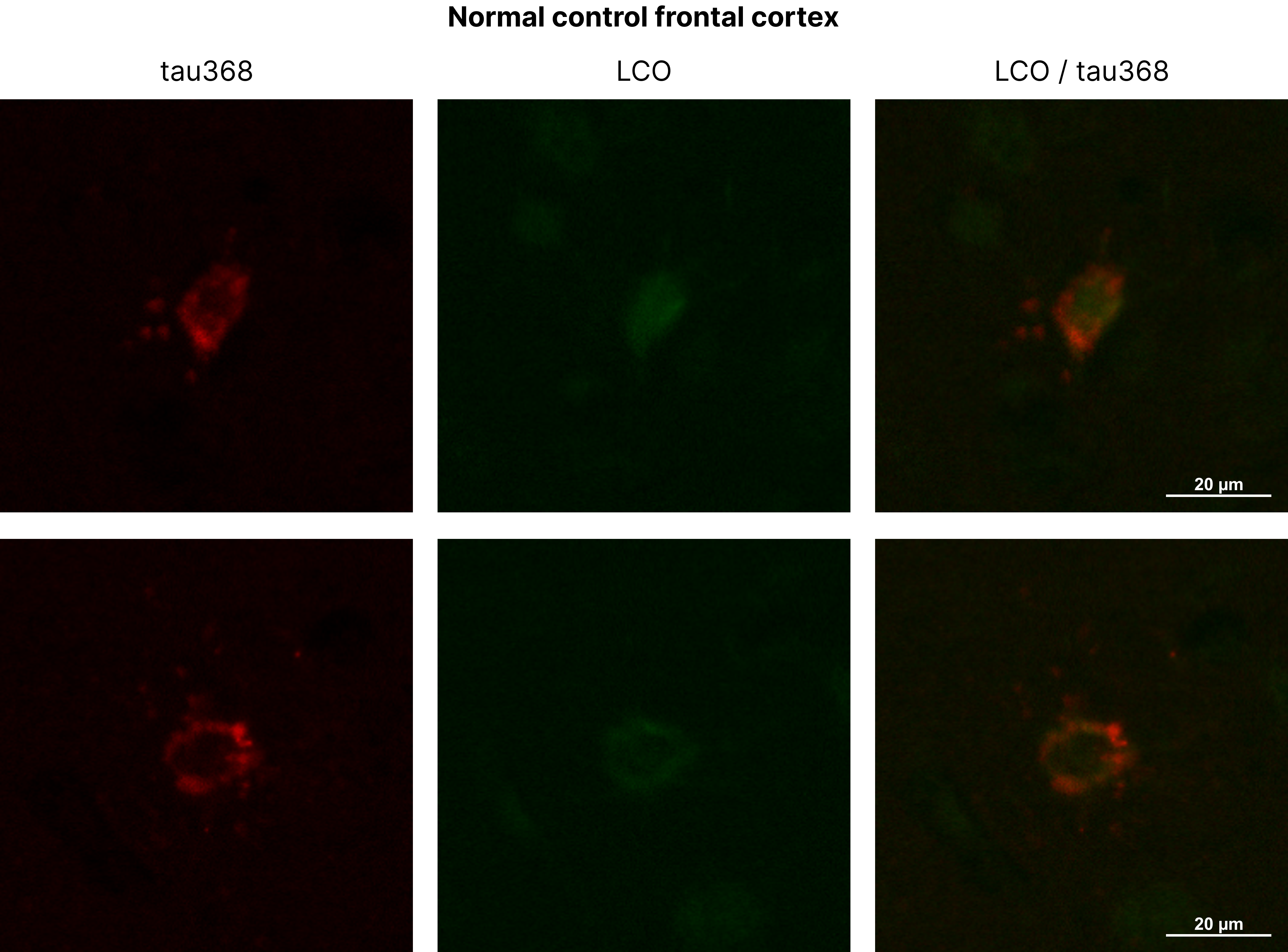
**Supplementary Figure 2. Tau368 and LCO staining in the normal control frontal cortex.**

Representative immunofluorescence images from normal control frontal cortex showing rare tau368-positive cells with faint staining and no detectable LCO signal. This finding supports the interpretation that sparse tau368 immunoreactivity may be present at low levels in control tissue without a corresponding LCO-positive fibrillar tau signal. Scale bars: 20 µm.


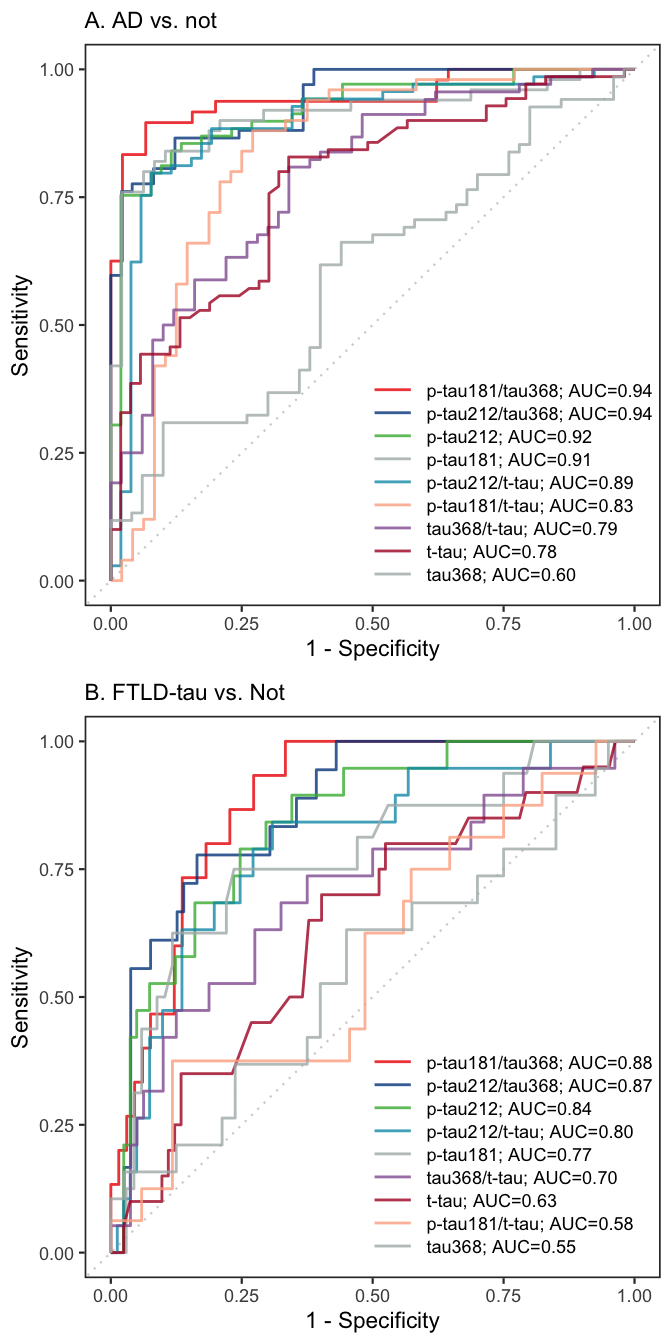


**Supplementary Figure 3. Receiver operating characteristic curves (ROC) to discriminate (a)** Alzheimer’s disease (AD) from other participants **(b)** discriminate FTLD-Tau from non-Tau participants.


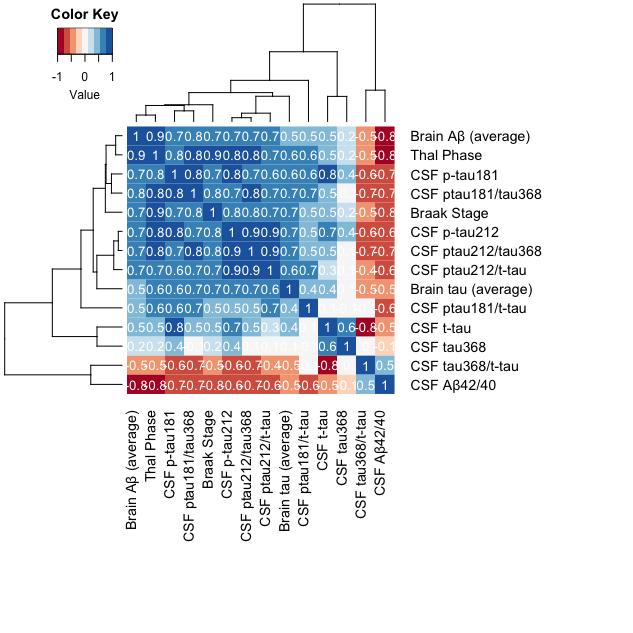


**Supplementary Figure 4. Correlation of tau biomarkers with ADNC and CSF Aβ42/40 ratio in the whole cohort.** The matrix presents Spearman correlations of tau biomarkers with Thal Phase, Braak Stage, Brain Tau (averaged over sampled regions), Brain Amyloid (averaged over sampled regions), and CSF Aβ42/40 ratio. Spearman’s rho is reported. White cells indicate p>0.05.


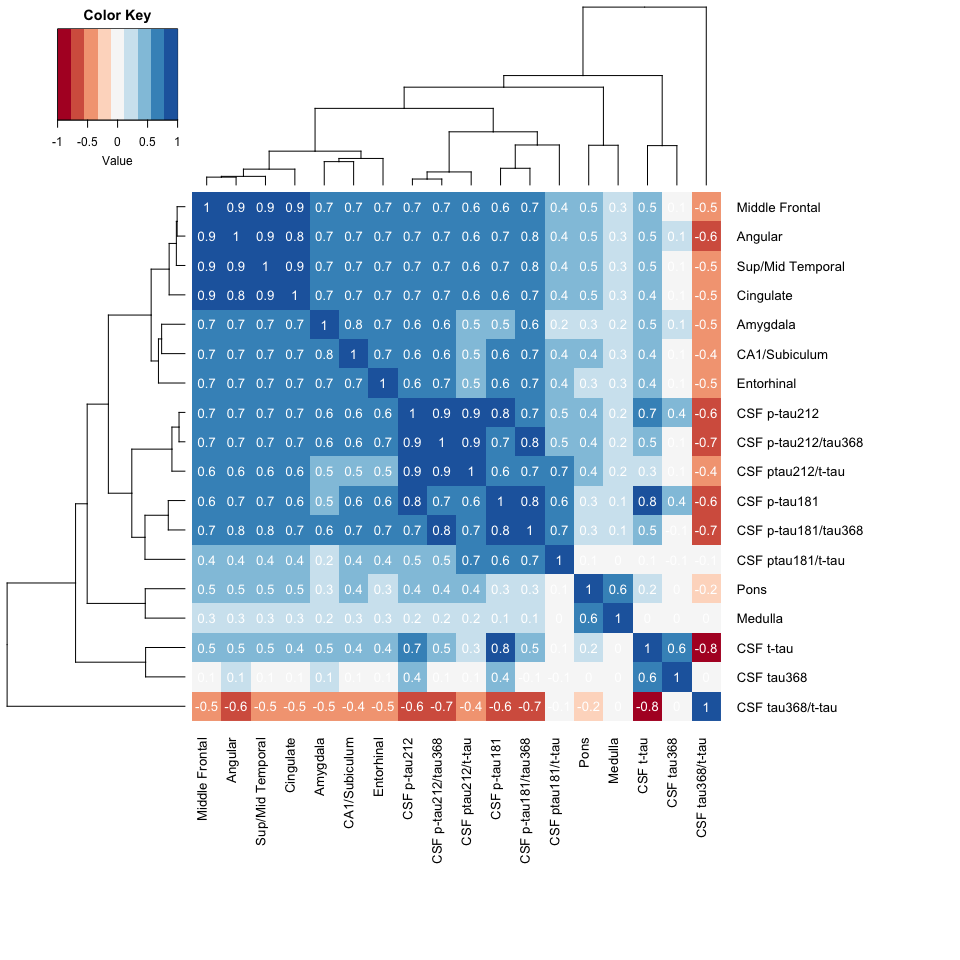


**Supplementary Figure 5. Correlations with regional brain tau burden in the whole cohort.** Spearman correlations tested associations between tau biomarkers and tau burden across the brain regions. Spearman’s rho is reported. White cells indicate p>0.05.


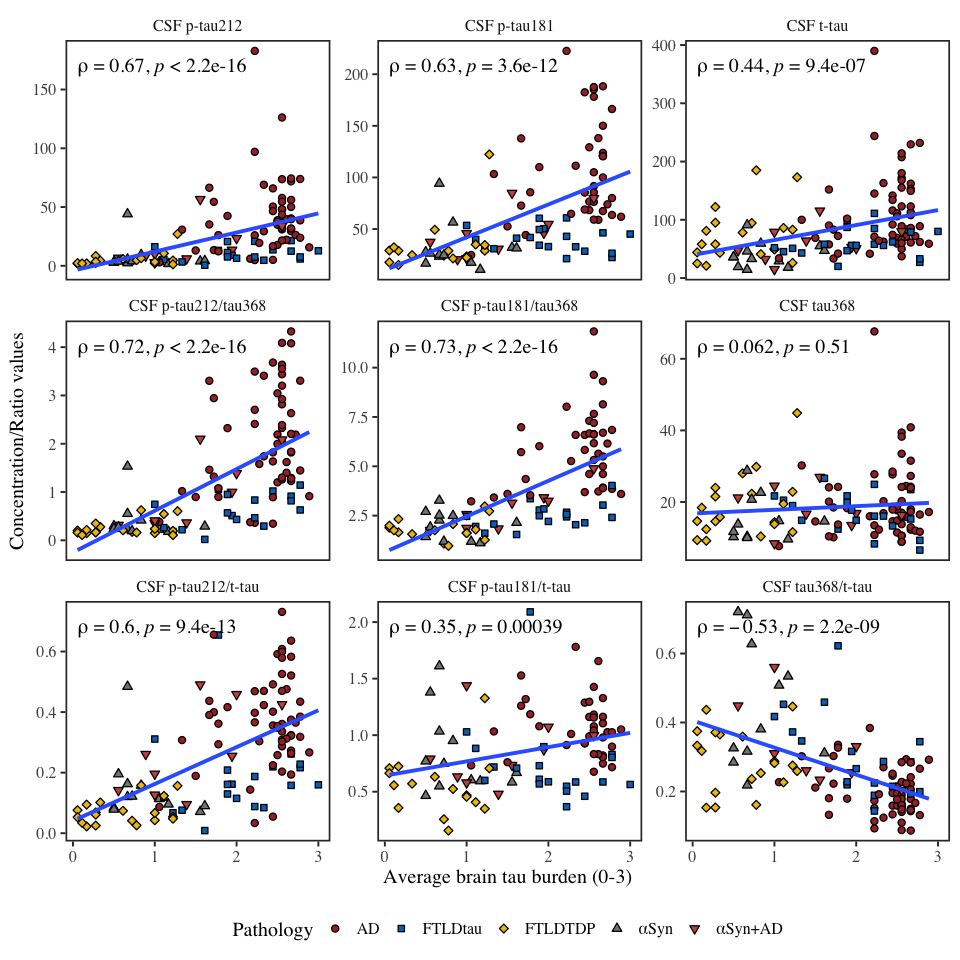


**Supplementary Figure 6. Average correlations of tau biomarkers with brain tau burden in whole cohort.** The Figure shows Spearman correlations between CSF biomarkers and average brain tau burden.


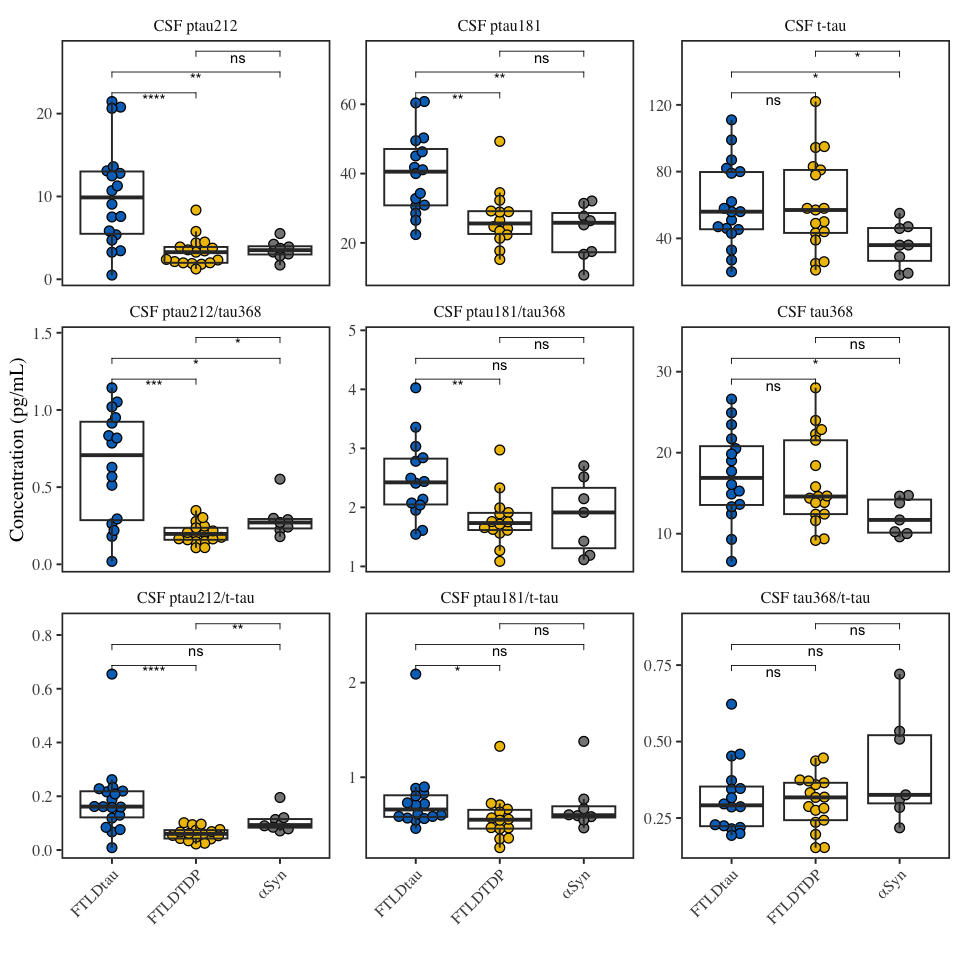


**Supplementary Fig 7. CSF tau biomarkers levels in ADNC-negative & Thal Phase < 2 neurodegenerative diseases.** The figure shows levels of tau biomarkers in FTLD-tau (n=18), FTLD-TDP (n=17) and αSyn (n=8) when intermediate and high ADNC and Thal Phases 2≥ are excluded. *; **; ***;**** represent p<0.05; p<0.01; p<0.001; p<0.0001 respectively.


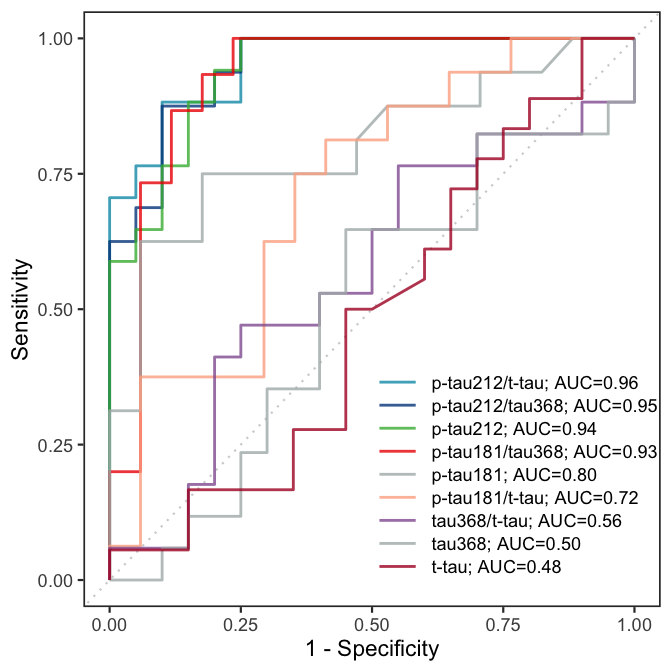


**Supplementary Fig. 8. Receiver operating characteristic curves (ROC) to discriminate FTLD-tau from other participants in ADNC-negative subcohort**. Excluding PSP (n=102).


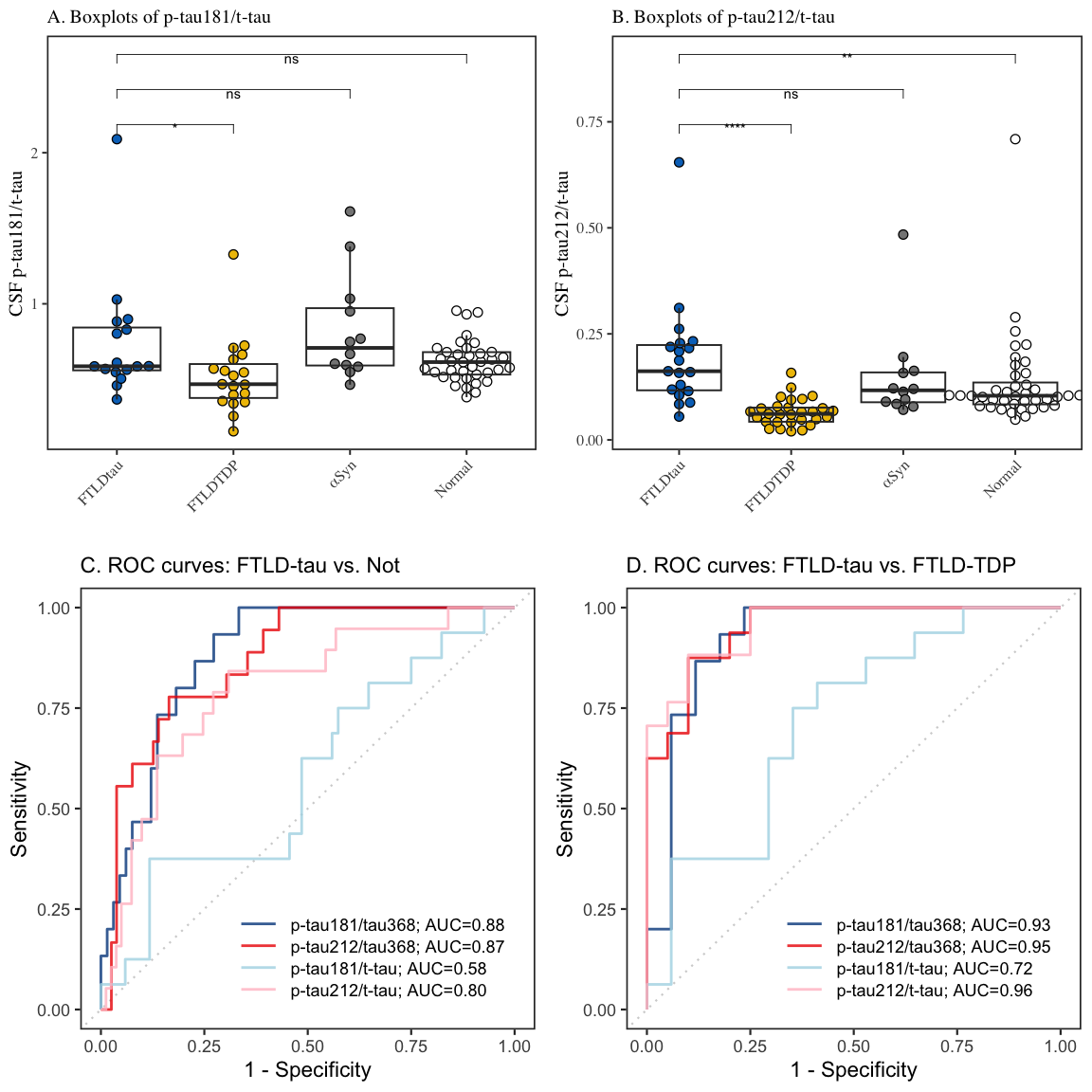


**Supplementary Fig. 9. Boxplots and receiver operating characteristic curves (ROC) of CSF p-tau181/t-tau and p-tau212/t-tau in ADNC-negative subcohort**. Excluding PSP (n=102).


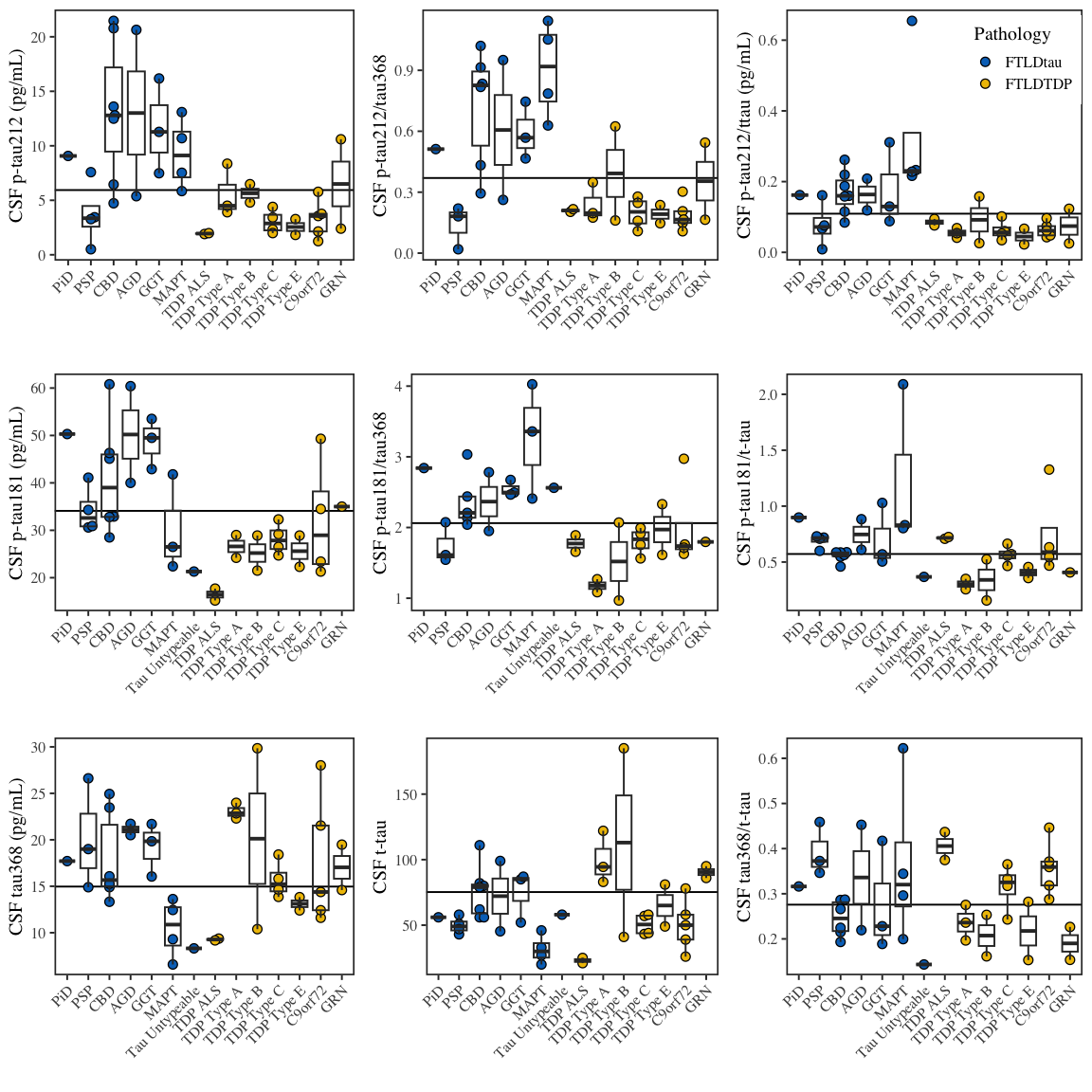


**Supplementary Figure 10. Levels of biomarkers in subtypes of FTLD-tau and FTLD-TDP**. The figure shows levels of p-tau212, p-tau212/tau368, p-tau181, p-tau181/tau368, p-tau212/t-tau, p-tau181/t-tau, tau368, and tau368/t-tau in FTLD-Tau (blue) neurodegenerative diseases: Pick’s Disease (PiD, n=1), progressive supranuclear palsy (PSP, n=4), corticobasal degeneration (CBD, n=7), argyrophilic grain disease (AGD, n=2), globular glial tauopathy (GGT, n=3), genetic tau mutations (MAPT, n=6) or untypeable tau pathology (n=1) and FTLD-TDP (yellow) neurodegenerative diseases: amyotrophic lateral sclerosis (ALS, n=2), FTLD-TDP type A (n=3), FTLD-TDP type B (n=2) FTLD-TDP type C (n=4), FTLD-TDP type E (n=2), participants with genetic mutation of C9orf72 (n=10), and participants with granulin mutation (GRN, n=5). Box plots represent median and interquartile range (IQR), and boundaries of the whiskers are minimum to maximum values. Horizontal lines indicate threshold to discriminate FTLD-tau from FTLD-TDP based on maximum Youden’s index, for p-tau212, p-tau212/tau368, p-tau181 and p-tau181/tau368.


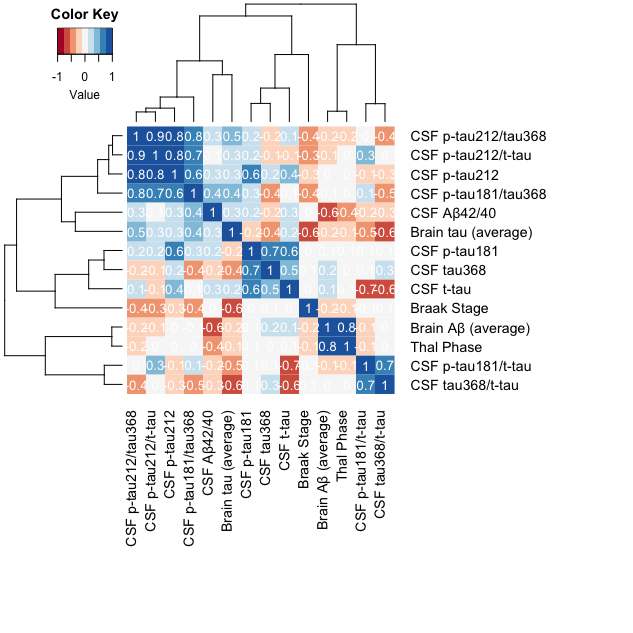


**Supplementary Figure 11. Correlation of tau biomarkers with ADNC and CSF Aβ42/40 ratio in ADNC-negative subcohort.** The matrix presents Spearman correlations of tau biomarkers with Thal Phase, Braak Stage, Brain Tau (averaged over sampled regions), Brain Amyloid (averaged over sampled regions), and CSF Aβ42/40 ratio. Spearman’s rho is reported.


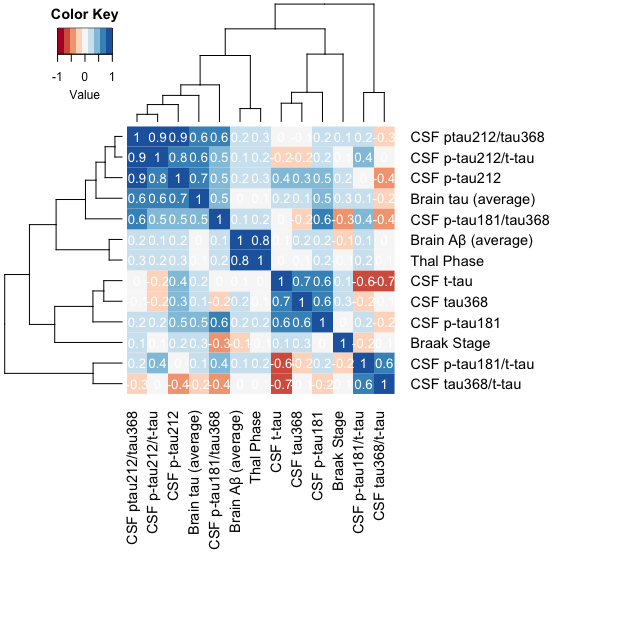


**Supplementary Figure 12. Correlation of biomarkers in ADNC-negative and Thal Phase<2 subcohort.** The matrix presents spearman correlations of tau biomarkers with ADNC. Spearman rho is reported.


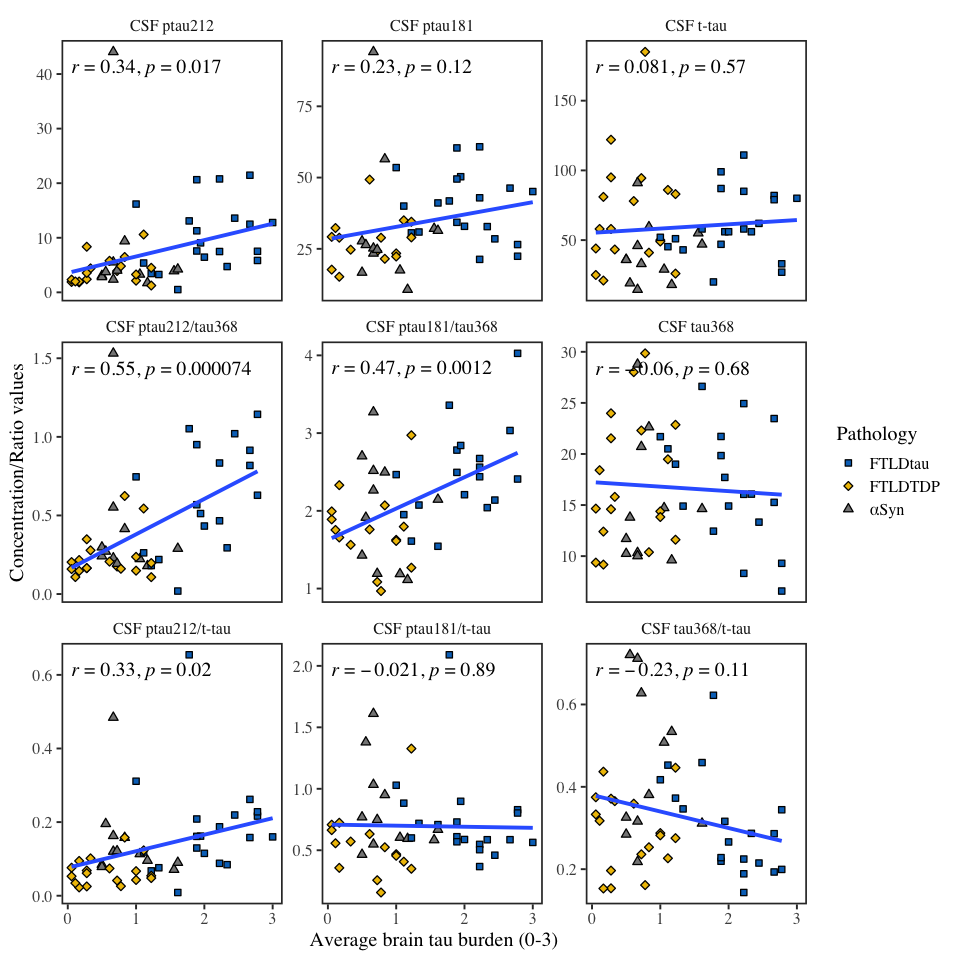


**Supplementary Figure 13. Average correlations of tau biomarkers with brain tau burden in ADNC-negative subcohort.** The figure shows Pearson’s correlations between CSF biomarkers and average brain tau burden.


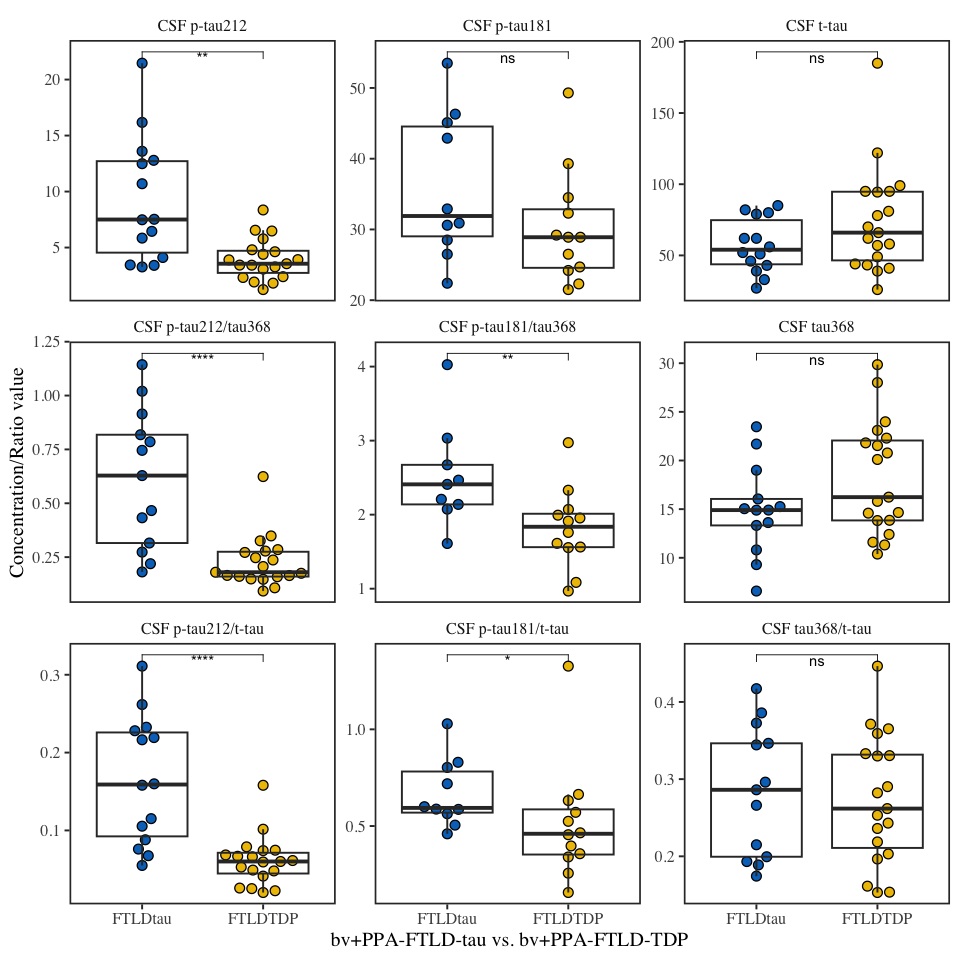


**Supplementary Figure 14. Levels of biomarkers in FTLD clinical variants, bvFTD and PPA.** The figure shows levels of tau biomarkers in clinically characterized behavioural variants of FTLD (bvFTD) and primary progressive aphasia (PPA) (bv+PPA-FTLD-tau (n=14); bv+PPA-FTLD-TDP (n=19). *; ** ; *** ; represent p<0.05; p<0.01; and p<0.001 respectively.


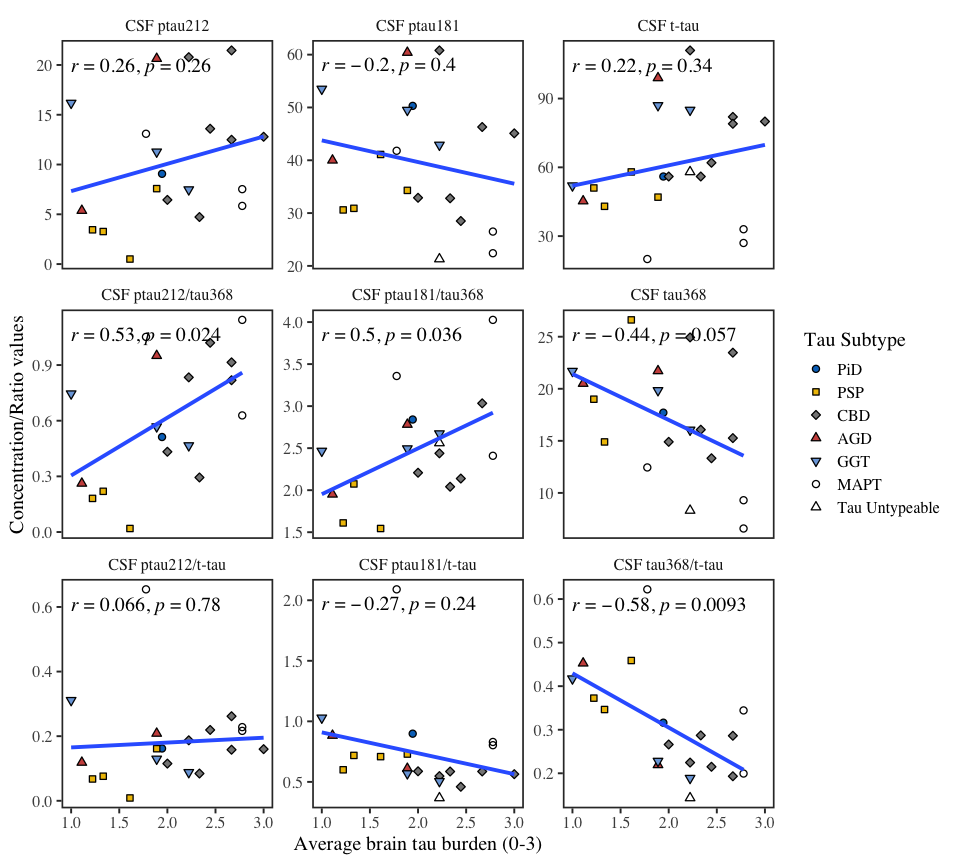


**Supplementary Figure 15. Average correlations of tau biomarkers with brain tau burden in ADNC-negative FTLD-tau group.** The Figure shows Pearson’s correlations between CSF biomarkers and average brain tau burden. Color indicates FTLD-tau molecular subtype.
